# Loss of *pex5* sensitizes zebrafish to fasting due to deregulated mitochondria, mTOR, and autophagy

**DOI:** 10.1007/s00018-023-04700-3

**Published:** 2023-02-23

**Authors:** Sushil Bhandari, Yong-Il Kim, In-Koo Nam, KwangHeum Hong, Yunju Jo, Kyeong-Won Yoo, Weifang Liao, Jae-Young Lim, Seong-Jin Kim, Jae-Young Um, Peter K. Kim, Ho Sub Lee, Dongryeol Ryu, Seok-Hyung Kim, SeongAe Kwak, Raekil Park, Seong-Kyu Choe

**Affiliations:** 1 Department of Medicine, Graduate School, Wonkwang University, Iksan, 54538 South Korea; 2 Sarcopenia Total Solution Center, Wonkwang University, Iksan, 54538 South Korea; 3 Institute of Brain Science, Wonkwang University, Iksan, 54538 South Korea; 4 Department of Molecular Cell Biology, Sungkyunkwan University School of Medicine, Suwon, 16419 South Korea; 5 Department of Microbiology, Wonkwang University School of Medicine, Iksan, 54538 South Korea; 6 Department of Biomedical Science, Graduate School, Wonkwang University, Iksan, 54538 South Korea; 7 Department of Pharmacology, College of Korean Medicine, Kyung Hee University, Seoul, 02447 South Korea; 8 Department of Biochemistry, University of Toronto, Toronto, ON M5S 1A8 Canada; 9 Hanbang Cardio-Renal Research Center, Wonkwang University, Iksan, 54538 South Korea; 10 Department of Biomedical Science and Engineering, Institute of Integrated Technology, Gwangju Institute of Science and Technology, Gwangju, 61005 South Korea; 11 Institute of Wonkwang Medical Science, Wonkwang University, Iksan, 54538 South Korea

**Keywords:** *pex5*, Zellweger spectrum disorder, Fasting, Mitochondria, mTOR, Autophagy

## Abstract

Animal models have been utilized to understand the pathogenesis of Zellweger spectrum disorders (ZSDs); however, the link between clinical manifestations and molecular pathways has not yet been clearly established. We generated peroxin 5 homozygous mutant zebrafish (*pex5*^−/−^) to gain insight into the molecular pathogenesis of peroxisome dysfunction. *pex5*^−/−^ display hallmarks of ZSD in humans and die within one month after birth. Fasting rapidly depletes lipids and glycogen in *pex5*^*−/−*^ livers and expedites their mortality. Mechanistically, deregulated mitochondria and mechanistic target of rapamycin (mTOR) signaling act together to induce metabolic alterations that deplete hepatic nutrients and accumulate damaged mitochondria*. *Accordingly, chemical interventions blocking either the mitochondrial function or mTOR complex 1 (mTORC1) or a combination of both improve the metabolic imbalance shown in the fasted *pex5*^−/−^ livers and extend the survival of animals. In addition, the suppression of oxidative stress by N-acetyl L-cysteine (NAC) treatment rescued the apoptotic cell death and early mortality observed in *pex5*^*−/−*^. Furthermore, an autophagy activator effectively ameliorated the early mortality of fasted *pex5*^*−/−*^. These results suggest that fasting may be detrimental to patients with peroxisome dysfunction, and that modulating the mitochondria, mTORC1, autophagy activities, or oxidative stress may provide a therapeutic option to alleviate the symptoms of peroxisomal diseases associated with metabolic dysfunction.

**Supplementary Information:**

The online version contains supplementary material available at 10.1007/s00018-023-04700-3.

## Introduction

Peroxisomes are highly dynamic and perform the breakdown of fatty acids, amino acids, and polyamines, biosynthesis of ether phospholipids, and scavenging of reactive oxygen species [1–3]. The importance of peroxisomal function in human health has been reported in numerous diseases, including peroxisomal biogenesis disorders, peroxisomal transporter or enzyme deficiencies, related metabolic disorders, aging-associated diseases, and cancers [4–8]. Functional peroxisomes require an array of peroxin (PEX) proteins, most of which reside in the peroxisome membrane, while few recycle between the cytosol and peroxisome membrane for the import of matrix proteins [1].

PEX5 acts as a major importing factor for peroxisome matrix proteins containing the C-terminal tripeptide (SKL) peroxisomal targeting signal, which is detected in the cytosol and transported to peroxisomes [9]. Accordingly, mutations in human *PEX5* cause Zellweger spectrum disorders (ZSDs) in which functional peroxisomes are largely absent [10]. Patients with Zellweger syndrome, the most severe form of ZSD, display hypotonia, craniofacial defects, neuronal hypomyelination, and hepatic and renal dysfunction and die within their first year of life [5]. Mice with homozygous *Pex5* mutations (*Pex5*^*−/−*^ mice) recapitulate the phenotype of Zellweger syndrome in humans [11, 12]. Because of the early postnatal death of *Pex5*^*−/−*^ mice, tissue-specific *Pex5*^*−/−*^ mice were generated and analyzed to further identify the molecular pathways leading to ZSD-like phenotypic presentation [11, 13]. Given that the liver is one of the most peroxisome-enriched organs [7, 14–16], liver-specific *Pex5*^*−/−*^ mice were found to be metabolically unsound, displaying a skewed nutrient preference toward carbohydrates with altered mitochondrial morphology and activity [11, 15, 17–19]. Similar to *Pex5* deficiency, deficient models of other *Pex* genes, such as *Pex2*, *Pex13*, *Pex16*, and *Pex19*, also show mitochondrial abnormalities [20–23]. These include reduced mitochondrial activity, increased reactive oxygen species (ROS), and structural impairment of the mitochondria, suggesting the presence of a fundamental mechanism dependent on peroxisome function that regulates mitochondrial homeostasis. Given the cooperative nature of peroxisomes and mitochondria in maintaining cellular homeostasis, peroxisome deficiency is understood to impact mitochondrial function, although how this is achieved remains unclear.

A recent report suggested that stress conditions, such as increased ROS, often found in metabolic disorders, could assemble tuberous sclerosis complex at the peroxisome, which represses the mechanistic target of rapamycin complex 1 (mTORC1) in cultured cells [24]. The same regulatory axis has also been suggested in cultured cells during starvation stress [25]. However, the stress-responsive regulatory axis involving peroxisomes is yet to be validated in vivo. For the discovery of peroxisome functions and participating players, various animal model systems, including yeast, flies, and mice, have been used by providing their unique advantage and, at the same time, by complementing the inherent drawbacks of each model system. The zebrafish is an established model for uncovering various aspects of biological processes and has recently been used in the field of peroxisome biology and associated human diseases. A pioneering study reported that peroxisomes in zebrafish are distributed in tissues in a manner similar to those in mammals [26]. More recently, we and other researchers have reported that zebrafish could serve as a model system for studying the metabolic role of peroxisome-resident proteins and the effects of their functional deficits using either the morpholino-mediated transient knockdown approach or transcription activator-like effector nucleases [27, 28]. Thus, zebrafish may bridge the gap between fly and mouse models in terms of evolutionary complexity, offering an opportunity to link functional interpretations obtained through genetic and developmental studies.

In this study, we aimed to characterize the role of peroxisomes in mitochondrial health and stress-induced regulatory responses. We generated a stable zebrafish model of ZSD by targeting the *pex5* gene. Zebrafish with homozygous *pex5* mutations (*pex5*^*−/−*^) share critical features of ZSD in humans. *pex5*^*−/−*^ zebrafish displayed significantly reduced amounts of peroxisomes and abnormal mitochondria at the cellular level, metabolic disturbances in the liver, and defective myelination in the nervous system at the tissue level. Notably, when fasting was imposed as an environmental stress, *pex5*^*−/−*^ rapidly developed phenotypic severity characterized by edema, deflated swim bladder, and decreased movement that could be easily detected through simple microscopy. Mechanistic studies have revealed a positive correlation between deregulated mitochondrial function in response to peroxisome dysfunction and unrepressed mTORC1 upon fasting. Chemical interventions, either mitochondrial function or mTORC1 signaling, rescued disturbed liver metabolism in fasted *pex5*^*−/−*^zebrafish. A similar rescue was observed with a ROS scavenger or an autophagy inducer. The identified pathway is suggested to be a druggable target for the treatment of symptoms in patients with ZSD.

## Materials and methods

### Fish maintenance, transgenic zebrafish, and generation of *pex5*^−/−^ zebrafish

All zebrafish lines were maintained at 28.5 °C. Embryos were collected and developed in E3 embryonic medium and staged according to age and morphology based on a standard protocol [29]. *pex5*^*−/−*^ zebrafish were generated using the CRISPR/Cas9 system and genotyped as described in detail in Supplementary Methods. Transgenic zebrafish lines *Tg*(*mbp:EGFP*) [30]*, Tg*(*Xla.Eef1a1:RFP-SKL*) [31], and *Tg*(*Xla.Eef1a1:mlsEGFP*) [32] have been previously described. *mbp:EGFP* transgenic line was crossed with *pex5* heterozygotes to obtain the *mbp:EGFP* transgenic line in a *pex5*^*−/−*^ background. Similarly, *Xla.Eef1a1:RFP-SKL* and *Xla.Eef1a1:mlsEGFP* transgenic lines were crossed with *pex5* heterozygotes separately to obtain *Xla.Eef1a1:RFP-SKL* and *Xla.Eef1a1:mlsEGFP* transgenic lines on *pex5*^*−/−*^ background, respectively.

### Microscopy

For whole-mount images of zebrafish larvae, the larvae were anesthetized using 1X tricaine and positioned using a 1.5% agarose plate. Images were taken using a Leica M165FC microscope equipped with Leica DFC500 (Leica, Wetzlar, Germany). Images of cryo-sectioned Oil Red-O (ORO)-stained, periodic acid-Schiff (PAS)-stained, and tunnel samples were taken using an Olympus IX71 fluorescence microscope (Olympus, Tokyo, Japan). Other fluorescent images after antibody staining or from transgenic zebrafish were captured using a Fluoview FV1000 confocal microscope (Olympus, Tokyo, Japan) or a ZEISS LSM 980 (Carl Zeiss AG, Oberkochen, Germany) at the Core Facility for Supporting Analysis & Imaging of Biomedical Materials at Wonkwang University, supported by the National Research Facilities and Equipment Center.

### Survival curve

Heterozygous *pex5*^+/−^ zebrafish were intercrossed and the resulting embryos were reared to the juvenile stage. From 15 days post-fertilization (dpf), dead larvae were collected and genotyped, and the ratio of each genotype was calculated assuming Mendelian segregation ratios. The surviving juveniles were fin-clipped, and their genotypes were determined. Three crosses were used to determine the survival rate (*n = *300).

### Cell/tissue staining

Immunofluorescence staining was performed as described in Supplementary Methods. ORO staining was performed as previously described [33]. In brief, larvae were fixed in 4% paraformaldehyde (PFA) overnight at 4 °C, washed with phosphate-buffered saline (PBS) three times, and infiltrated with a graded series of propylene glycol (PG) (i.e., 25% PG + 75% PBS, 50% PG + 50% PBS, 75% PG + 25% PBS, 100% PG) for 10 min. The larvae were then incubated with 0.5% freshly prepared ORO solution in 100% PG overnight at room temperature on a shaker and rinsed several times with PBS. Stained larvae were processed for cryo-sectioning to obtain 50-µM-thick section. Stained images of the liver tissue sections were captured using a Leica M165FC microscope equipped with a Leica DFC500 microscope. PAS staining was performed as previously described [33]. Larvae were fixed in 4% PFA overnight at 4 °C, washed with PBST three times, and permeabilized in 40 µg/mL proteinase K for 30 min at room temperature. Samples were then washed thrice in PBT, rinsed in demineralized water (DW), and oxidized in 0.5% periodic acid for 30 min at room temperature. After washing with DW three times for 20 min each, the larvae were stained with Schiff reagent for 5 min, followed by immediate rinsing in tap water. Larvae were then processed for cryo-sectioning to obtain a 50-µm-thick liver section. Liver tissues were mounted using coverslips and mounting solution. Images were acquired using an IX71 microscope (Olympus).

### Quantification of mitochondrial DNA and mitochondrial number

Wild-type (WT) and *pex5*^*−/−*^ livers were dissected from 6-day-old larvae using forceps and processed for genomic DNA extraction using the TIANamp Genomic DNA Kit (TIANGEN, Beijing, China) according to the manufacturer’s instructions. The remaining larvae were used for genotyping. For quantification of mitochondrial DNA, 15 ng of genomic DNA from each sample was amplified via polymerase chain reaction (PCR) using Fast Start Essential DNA Green Master (Roche, Basel, Switzerland), and the ratio of mitochondrial DNA to nuclear DNA was calculated. Primers used to amplify mitochondrial DNA and nuclear DNA were Mt-ND1 (F:5ʹ-CCCCAGATGCACCTGAGCTAATAAC-3ʹ and R:5ʹ- TGTTTGTGGGGGTAGACCAGCTAG-3ʹ) and β-actin (F:5ʹ-ACCTCATGAAGATCCTGACC-3ʹ and R:5ʹ-TGCTAATCCACATCTGCTGG-3ʹ). The data were analyzed to determine the relative abundance of *β-actin*. Mitochondrial numbers were manually counted. Three image fields were obtained for both WT and mutant fish using a confocal microscope (Olympus, Tokyo, Japan). All analyses were performed in triplicates.

### 3ʹUTR RNA-seq and data processing

Total RNA from fasted WT and *pex5*^−/−^ livers at 6 dpf was extracted using the Direct-zol RNA Kit (ZYMO RESEARCH, Irvine, CA, USA), according to the manufacturer’s instructions. 3'UTR RNA-seq was performed using the CEL-Seq2 protocol [34], except that the Second Strand Synthesis Module (NEB, Ipswich, MA, USA) was used for double-stranded cDNA synthesis, and the library was amplified by nine cycles of PCR without any sample pooling. The library was sequenced on an Illumina MiniSeq, and the following quantitative analysis was performed using 51 bp of insert reads (Read2). Cell barcode and UMI in Read1 was extracted by using UMI-tools (ver.1.1.1) with the following command “umi_tools extract -I read1.fastq --read2-i*n = *read2.fastq --bc-pattern = =NNNNNNNNNNCCCCCCCCC.” Four cell barcodes were mixed and used for all the samples. Adaptor sequences and low-quality sequences were removed, read length below 20 bp was discarded using Trim Galore (ver.0.6.7), and reads were mapped to the GRCz11 reference using HISAT2 (ver.2.2.1). Read counts for each gene were obtained using featureCounts (ver.2.0.1), and UMI duplications were removed using UMI-tools. To further analyze and visualize the gene expression profiles, the processed count was transformed into a transcript per million (TMM) using the edgeR package (ver.3.36.0) as described previously [35]. R Studio (ver.2022.02.2) and R (ver.4.1.2) were used to create scatter plots showing gene expression profiles (log_2_[TMM + 1]) and (2) differential gene expression (log_2_[fold change]). R included the R packages dplyr (ver.1.0.9), stringr (ver.1.4.0), ggpubr (ver.0.4.0), ggplot2 (ver.3.3.6), and pheatmap (ver.1.0.12).

### mRNA expression

WT and *pex5*^*−/−*^ livers were dissected from six- or 7-day-old larvae. Each sample of total RNA was prepared from ten livers at the desired developmental stages using TRIzol reagent (Ambion Inc., Austin, TX, USA), following the manufacturer’s instructions. Total RNA was reverse-transcribed using reverse transcriptase (Roche, Basel, Switzerland), and quantitative PCR (qPCR) was performed using the following primers: *p62* F:5ʹ-GCGTCAGTGAGGGAACAAAG-3ʹ, R:5ʹ-CAGAGACTCCACCAGCCTAG-3ʹ; and *β-actin* F:5ʹ-TGAATCCCAAAGCCAACAGAGAGA-3ʹ, R:5ʹ-TCACGACCAGCTAGATCCAGACG-3ʹ. The data were analyzed to determine the relative abundance of *β-actin*. All analyses were performed in triplicate.

### Transmission electron microscopy (TEM)

WT and *pex5*^*−/−*^ livers were dissected from 4.5- and 6.5-day-old embryos. Dissected zebrafish livers were fixed with 2% paraformaldehyde and 2% glutaraldehyde in 0.05 M sodium cacodylate buffer (pH 7.2) overnight at 4 °C. Fixed samples were washed three times with 0.05 M sodium cacodylate buffer for 10 min at 4 °C and post-fixed with 1% osmium tetroxide in 0.05 M sodium cacodylate buffer. The post-fixed samples were washed twice with distilled water for 10 min at room temperature. The samples were then stained in en bloc for overnight at 4 °C. The samples were then dehydrated in a graded ethanol series, followed by propylene oxide, and embedded in Quetol-Spurr resin. Sections (70 nm thick) were cut on a Leica Ultracut ultramicrotome (Leica Microsystem, EM UC7, Canada), stained with 2% uranyl acetate and lead citrate, and viewed under a Hitachi H-7650 TEM (Hitachi Science and Technology, Japan).

### Cell death assay

Larvae were processed for cryo-sectioning to obtain a 10-µm-thick liver section, dried for 1 h at room temperature, and washed with PBS three times for 10 min. The samples were incubated in blocking solution (3% hydrogen peroxide (H_2_O_2_) in methanol) for 10 min at room temperature, followed by washing with PBS. Permeabilization solution (10 µL 1 M Tris–HCl pH 7.5 + 2 µL proteinase K PCR Grade 20 mg/mL and H_2_O to make 1 mL) was then added to the tissue samples, incubated for 2 min at 4 °C, and the slides were rinsed with PBS. As the positive control, fixed and permeabilized samples were incubated with DNase (20 U/mL DNase in 50 mM Tris–HCl pH 7.5, 10 mM magnesium chloride (MgCl_2_), and 1 mg/mL bovine serum albumin) for 10 min at room temperature, followed by washing with PBS twice. The area around the tissue was then dried, and 50 µL terminal deoxyribonucleotidyl transferase (TDT)-mediated dUTP nick-end labeling (TUNEL) reaction mixture (50 µL enzyme solution + 450 µL label solution, Roche, Basel, Switzerland) was added to the tissue sample, followed by incubation for 60 min at 37 °C in a humidified atmosphere in the dark. The slides were washed three times in PBS, and 4ʹ, 6-diamidino-2-phenylindole (DAPI, Roche 10236276001, Basel, Switzerland) (1:1000 in PBS) was added to the tissue samples and incubated for 10 min at room temperature in the dark. Samples were then washed three times in PBS, mounted with a clear mount, and imaged under an Olympus IX71 fluorescence microscope.

### Drug treatment

For rescue experiments, rapamycin (250 nM) or UK5099 (25 µM) was treated in embryo medium starting from 3.5 dpf, and etomoxir (1.5 µM), oligomycin (5 pM), chloroquine (250 μM), or N-acetyl-cysteine (NAC) (100 μM) was treated from 5 dpf. The medium containing the drug was changed daily until the analysis.

### Immunoblotting

After genotyping, WT and *pex5*^−/−^ at 6 dpf were separately pooled in a 1.5 ml Eppendorf tube and sonicated on ice using 1 mL PBS to break embryos into pieces. Samples were centrifuged at 12,000 rpm for 5 min, and lysis buffer containing protease inhibitor cocktail was added to the pellets and incubated on ice for 1 h. Samples were then centrifuged at 14,000 rpm at 4 °C, and the supernatant was mixed with sodium dodecyl sulfate (SDS) loading dye and heat shock at 95 °C for 5 min. Samples were then loaded on a sodium dodecyl sulfate–polyacrylamide gel electrophoresis (SDS-PAGE) gel and run in running buffer for 2 h at 100 V. The proteins were transferred to a nitrocellulose membrane using a transfer buffer for 1.5 h at 40 V. The membrane was then blocked for 1 h at room temperature using blocking buffer (5% skim milk in 1X TBST). The membrane was then incubated with primary antibody diluted in blocking buffer (2.5% skim milk in TBST) overnight at 4 °C and washed thrice with TBST for 10 min. Secondary antibodies diluted appropriately in blocking buffer were then added to the membrane (2.5% skim milk in TBST) and incubated at room temperature for 1 h, followed by three washes with TBST for 10 min each. All washes and antibody incubations were performed using a rocker. For signal development, all buffers were removed and the membrane was incubated for 3 min in a western blot detection kit (AbFrontier, Seoul, South Korea) and developed in a dark room using an X-ray film, developer, and fixer. After the signal development, the X-ray film was washed in running water for a few minutes, and images were captured.

### Antibodies

Antibodies used for western blotting or immunostaining in this study are listed in Supplementary Methods.

### Statistics

Statistical significance was determined using the Student’s *t* test in Microsoft Excel; * and ** indicate *p* values < 0.05 and < 0.01, respectively.

### Data availability

The RNA-seq dataset used in this study was deposited in the Gene Expression Omnibus and is available under accession number GSE205349.

### Study approval

The experimental protocol was approved by the Committee for Ethics in Animal Experiments of Wonkwang University (WKU15-126) and was performed in accordance with the guidelines for animal experiments.

## Results

### *pex5*^−/−^ zebrafish recapitulates clinical characteristics of ZSD

**Fig. 1:**
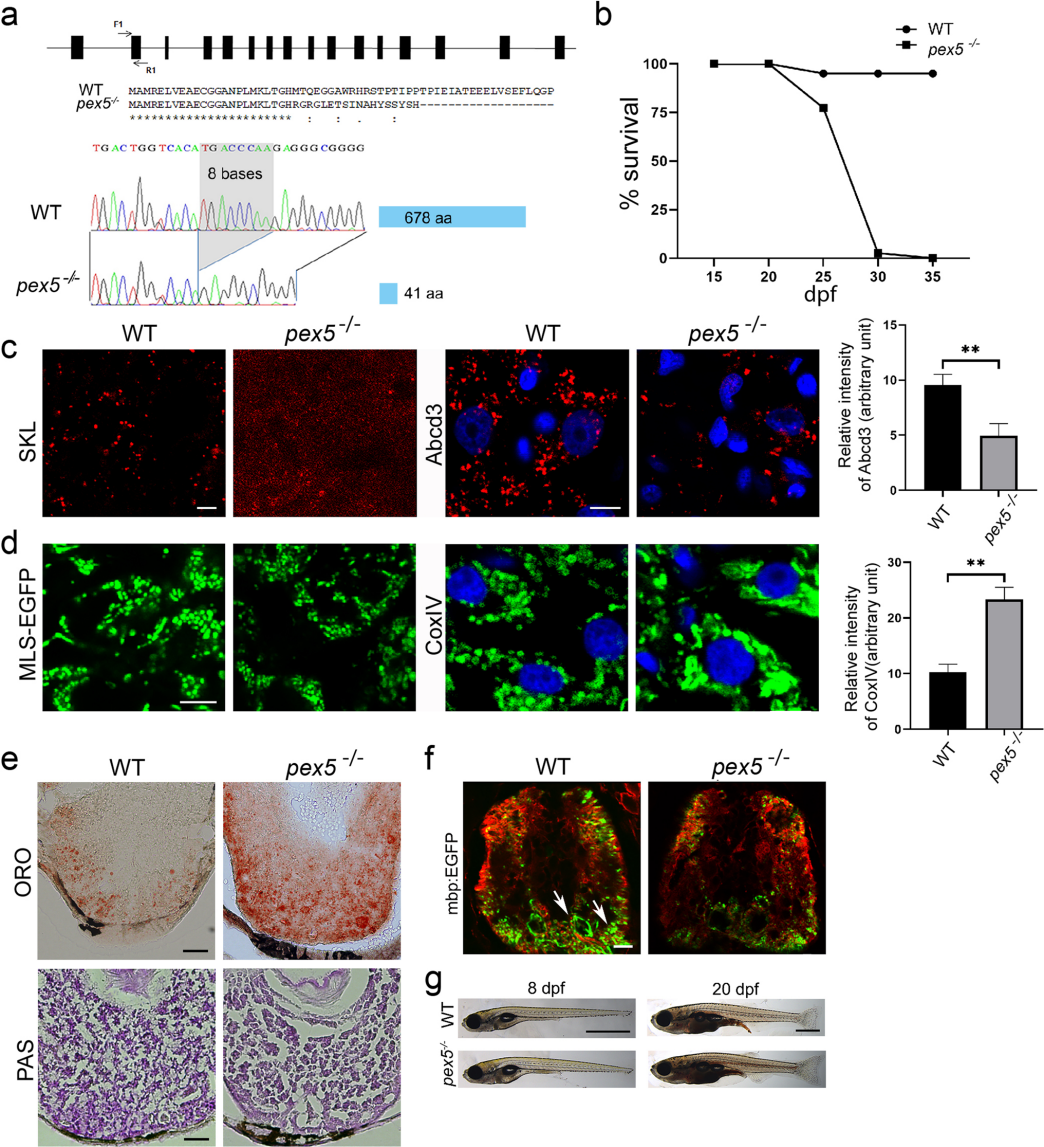
Loss of Pex5 in zebrafish induces liver defects and myelination abnormality due to the decreased peroxisome number. **a** Schematic view of the genomic structure of *pex5* in zebrafish is shown with 16 exons in black rectangles. F1 and R1 indicate the position and direction of the primer sequences that were used for the clustered regularly interspaced palindromic repeat/caspase 9 (CRISPR/Cas9)-mediated gene inactivation. A partial amino-terminal sequence of WT Pex5 is compared to that of Pex5 mutant protein. Note that the 8-base pair deletion shown in the sequencing chromatogram of the *pex5* gene is predicted to translate a truncated version of the Pex5 mutant protein comprised only 41 amino acids with a stretch of unrelated carboxy-terminal sequences. **b** Survival curve for *pex5*^*−/−*^ under normal feeding conditions. Heterozygous *pex5*^+/−^ zebrafish were incrossed, and the percentage of survived larvae per genotype at the indicated developmental times was plotted with assumption of Mendel’s segregation ratios. **c** Peroxisomes are shown in the liver sections of WT and *pex5*^*−/−*^. The RFP-SKL expression from the *Xla.Eef1a1:RFP-SKL* transgene was used as a surrogate to detect the subcellular localization of the peroxisomal matrix proteins. The expression levels of endogenous PMP70 proteins detected by immunofluorescence were shown to illustrate an abundance of peroxisome membrane proteins that are independently incorporated in the peroxisome membrane from the matrix-import pathway. Scale bar = 5 μm. Graph shows quantified signal intensity of Abcd3 and presented as the average with error bars indicating standard deviation. Statistical significance was determined using the Student’s *t* test in Microsoft Excel; * and ** indicate *p* values < 0.05 and < 0.01, respectively. **d** Mitochondria are shown in the liver sections of WT and *pex5*^*−/−*^. Expression of mitochondria-targeted enhanced green fluorescent protein (EGFP) (MLS-EGFP) was used to detect the general shape of mitochondria, whereas immunofluorescence of a component of the mitochondrial respiratory chain complex IV (CoxIV) was performed to reveal the differential expression of an endogenous mitochondria-targeted protein. Scale bar = 5 μm. Graph shows quantified signal intensity of CoxIV and presented as the average with error bars indicating standard deviation. Statistical significance was determined using the Student’s *t* test in Microsoft Excel; * and ** indicate *p* values < 0.05 and < 0.01, respectively. **e** Neutral lipids and glycogen are shown by performing Oil Red O and PAS staining, respectively, in the liver sections of WT and *pex5*^*−/−*^. Scale bar = 50 μm. **f** Neuronal myelination using *Tg(mbp:EGFP)* transgenic zebrafish was compared between WT and *pex5*^*−/−*^. Red immunofluorescence for acetylated tubulin was used to locate the differentiating neurons in the zebrafish trunk. Arrows indicate myelinated neurons. Scale bar = 50 μm. **g** Representative pictures of the WT and *pex5*^*−/−*^ at 8 and 20 dpf as indicated are shown in lateral views with anterior to the left. Scale bar = 1 mm

To establish a stable zebrafish model for ZSD, we initially examined the equivalence of the zebrafish *pex5* gene with its mammalian counterpart. Sequence analysis suggested that PEX5 is highly conserved among vertebrates including humans, mice, and zebrafish (Supplementary Fig. S1). To address the functional equivalence of *PEX5* genes, we generated *pex5* mutant zebrafish using the CRISPR-Cas9 system, which targeted an optimal genomic sequence near intron/exon junction 2 (Fig. 1a). A founder zebrafish harboring an 8-base pair deletion in the *pex5* open reading frame was identified and used to produce *pex5* F1 heterozygous zebrafish, whose *pex5* gene was predicted to generate a truncated version of the protein comprising only 41 amino acids (Fig. 1a). Almost all *pex5*^*−/−*^ generated from the incrosses of *pex5* heterozygotes died before one month after fertilization (Fig. 1b).

To address the mechanistic aspects of *pex5*^*−/−*^ mortality, we initially examined peroxisomes that were predicted to be dysfunctional in zebrafish *pex5*^*−/−*^, as observed in human patients or animal models with *PEX5* deficiency [36, 37]. Expression of RFP-SKL (a cargo protein of Pex5 with red fluorescence) from the *Xla.Eef1a1:RFP-SKL* transgene [27] showed subcellular distribution of peroxisomes in a punctate pattern in the hepatocytes of WT zebrafish at 20 days post-fertilization (dpf) (Fig. 1c, *n* = 6). In contrast, RFP-SKL was diffusely detected in the cytoplasm of *pex5*^*−/−*^ hepatocytes, which is consistent with the Pex5 deficiency in the import of peroxisome matrix proteins in mammals. Moreover, a decreased number of punctate structures expressing Abcd3 (*n = *6), an endogenous peroxisome membrane protein localized in a Pex5-independent manner, indicated a decreased number of peroxisomes at 20 dpf in the *pex5*^*−/−*^ livers.

We next examined mitochondria, whose abnormalities have been reported in ZSD [11, 17, 18, 36]. Mitochondrial morphologies in the hepatocytes of WT zebrafish, assessed by the expression of green fluorescent protein fused to mitochondria-localizing signal (MLS-EGFP) from Tg(*Xla.Eef1a1:MLS-EGFP*) transgenic zebrafish [32], differed from those in *pex5*^−/−^ whose mitochondria were heterogeneous in size and occasionally clustered (Fig. 1d, *n = *6). In addition, the expression of endogenous proteins belonging to the mitochondrial respiratory chain complex IV was highly increased in the *pex5*^*−/−*^ hepatocytes compared to that in WT (*n = *6), suggesting that mitochondrial abnormalities might have initiated at the subcellular levels earlier than 20 dpf.

ZSD features metabolic disturbances in the liver, the most peroxisome-rich organ [18, 36]. Indeed, we found that liver lipids were highly accumulated in *pex5*^*−/−*^ compared to those in WT, while glycogen levels between the two genotypes were comparable (Fig. 1e, *n* = 6). ZSD also features defects in the nervous system, including demyelination [38, 39]. Using transgenic zebrafish, *Tg*(*mbp:EGFP*), we found that *pex5*^−/−^ zebrafish at 20 dpf displayed severe demyelination (Fig. 1f, *n* = 6). Despite the metabolic alterations in the liver and neuronal demyelination, the resulting *pex5*^*−/−*^ did not show noticeable differences in gross morphology from WT siblings (Fig. 1g, *n* > 40), but died before one month of age (Fig. 1b). Given that the molecular and cellular phenotypes and early mortality displayed in *pex5*^*−/−*^ resembled those in ZSD, this model could be used as another system to identify the molecular pathways underlying ZSD pathogenesis.

### *pex5*^−/−^ zebrafish are sensitized to fasting leading to expedited death during the larval stage

**Fig. 2:**
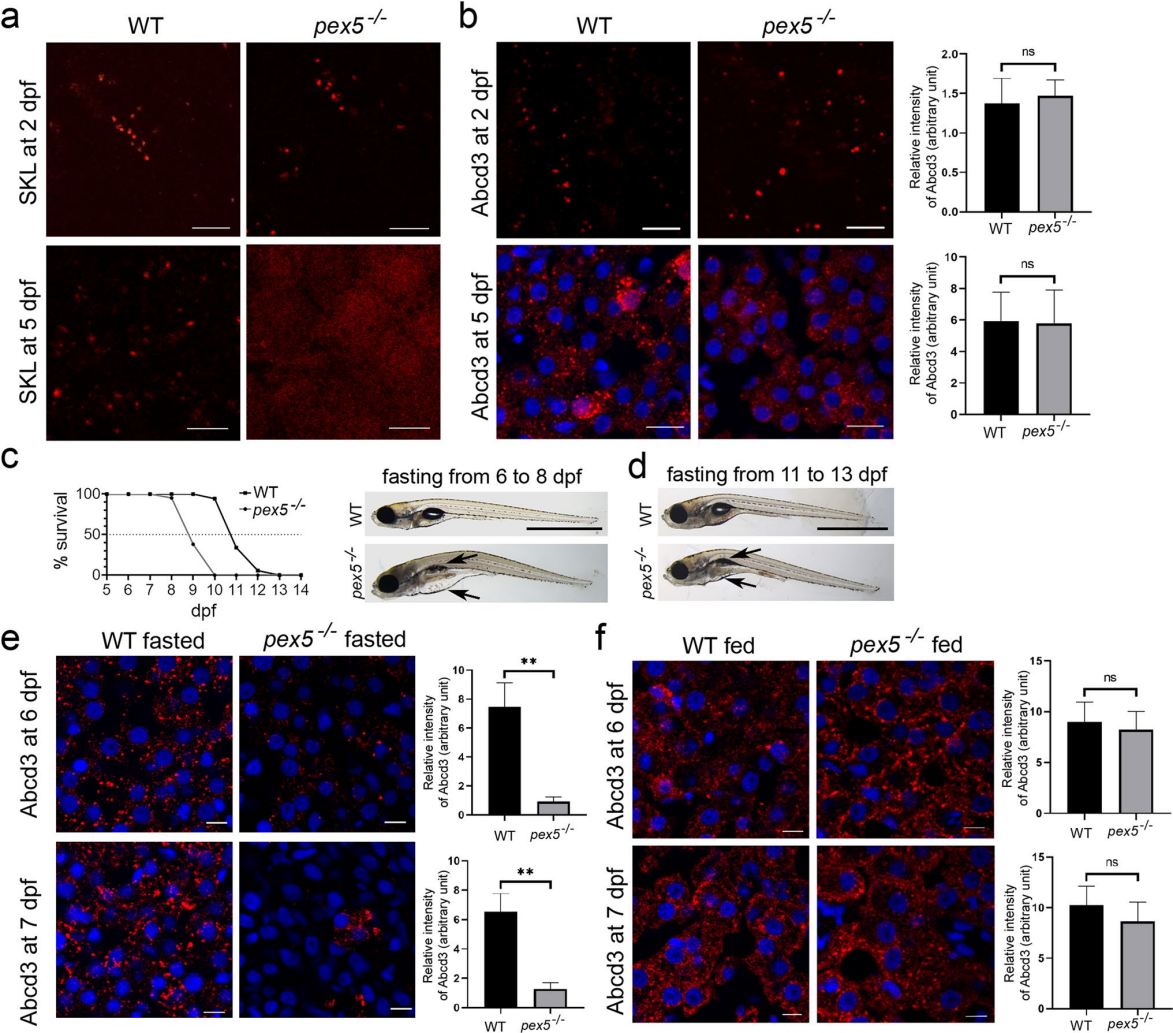
Fasting induces the rapid exacerbation of *pex5*^*−/−*^ leading to early mortality. **a** RFP-SKL was detected using the *Xla.Eef1a1:RFP-SKL* transgenic zebrafish at 2 dpf in the muscle cells and at 5 dpf in the liver sections of WT and *pex5*^*−/−*^. Note that the SKL signal is diffusely detected in the liver cells of *pex5*^*−/−*^ at 5 dpf. Scale bar = 5 μm. **b** Endogenous Abcd3 proteins were shown by performing immunofluorescence at 2 dpf in the muscle cells and at 5 dpf in the liver sections of WT and *pex5*^*−/−*^. Scale bar = 5 μm. Graphs show quantified signal intensity of Abcd3 at the indicated times and presented as the average with error bars indicating standard deviation. Statistical significance was determined using the Student’s *t* test in Microsoft Excel. **c** Survival curve shows a differential survival response between the WT and *pex5*^*−/−*^ zebrafish under fasting conditions. Experiments were performed at least three times and the average percentage of surviving animals was used to draw the curve (*n = *40/genotype). Representative pictures of the WT and *pex5*^*−/−*^ fasted from 5 to 8 dpf are shown in lateral views with anterior to the left. Arrows point to the edematous gut area and deflated swim bladder in fasted *pex5*^*−/−*^ at 8 dpf. Scale bar = 1 mm. **d** Representative pictures of WT and *pex5*^*−/−*^ fasted from 11 to 13 dpf are shown as indicated. Scale bar = 1 mm. **e**, **f** Peroxisome abundance was compared in the liver cells between the WT and *pex5*^*−/−*^ and between the feeding and fasting conditions using immunofluorescence for Abcd3. There was a marked disappearance of peroxisome abundance depending on the *pex5* genotype and the nutritional status. Scale bar = 5 μm. Graphs show quantified signal intensity of Abcd3 under fed or fasted conditions at the indicated times and presented as the average with error bars indicating standard deviation. Statistical significance was determined using the Student’s *t* test in Microsoft Excel; ** indicates *p* values < 0.01

Although *pex5*^−/−^ zebrafish could survive up to one month of age, *pex5* deficiency might have altered organelle structures and, thus, their functions as early as 20 dpf. To trace the stage in which peroxisomes became dysfunctional in *pex5*^−/−^ zebrafish, we examined the expression of both RFP-SKL and Abcd3 during early embryonic development in zebrafish. Owing to the maternal contribution of peroxisomes and *pex5* gene products, RFP-SKL was still detected in a punctate structure in *pex5*^−/−^ at 2 dpf. However, as peroxisome turnover is predicted to be approximately two days on average [40], RFP-SKL was diffusely detected in the cytosol of the hepatocytes of *pex5*^−/−^ zebrafish at 5 dpf (Fig. 2a, *n* = 8). Notably, the number of peroxisomes detected by Abcd3 antibody staining was similar in WT and *pex5*^−/−^ zebrafish at 5 dpf (Fig. 2b, *n* = 8). These results suggest that peroxisomes in *pex5*^*−/−*^ might be immature as early as 5 dpf as opposed to those in WT and become reduced in number before 20 dpf.

Zebrafish embryos consume yolk for the initial five days of development, but depend on the external food supply for their development and growth [41, 42]. We reasoned that metabolic challenges might alter the phenotypic presentation of *pex5*^*−/−*^. We found that WT zebrafish subjected to fasting could survive up to 12 dpf, with 50% mortality observed at approximately 10.5 dpf (Fig. 2c, *n* = 40). In contrast, *pex5*^*−/−*^ zebrafish under the same condition displayed clearly observable phenotypes including edematous abdomen with deflated swim bladder, shrunken liver, and hypotonia at 8 dpf and died between 8 and 10 dpf (with 50% mortality observed at approximately 8.5 dpf, *n = *40), suggesting a high sensitivity to fasting. The causal relationship between fasting and early mortality of *pex5*^*−/−*^ was confirmed by performing another fasting experiment in which *pex5*^*−/−*^ subjected to fasting from 11–13 dpf recapitulated the phenotype and early mortality observed in *pex5*^*−/−*^ subjected to fasting from 6 dpf (Fig. 2d, *n* = 20).

We next examined the effect of fasting on peroxisomal abundance. In contrast to the comparable numbers of immature peroxisomes present in both WT and *pex5*^*−/−*^ livers at 5 dpf (Fig. 2b), fasting dramatically reduced peroxisome numbers in the hepatocytes of *pex5*^*−/−*^ at 6 dpf and almost completely reduced peroxisome numbers in most cells at 7 dpf (Fig. 2e, *n* = 8). However, peroxisome numbers under feeding conditions were comparable between the WT and *pex5*^*−/−*^ (Fig. 2f, *n* = 8). These results suggest that fasting-mediated metabolic challenge could discriminate functional peroxisomes from immature ones, whose disappearance was facilitated in fasted *pex5*^*−/−*^larvae.

### *pex5* depletion induces alteration in the hepatic mitochondria under fasting conditions

**Fig. 3:**
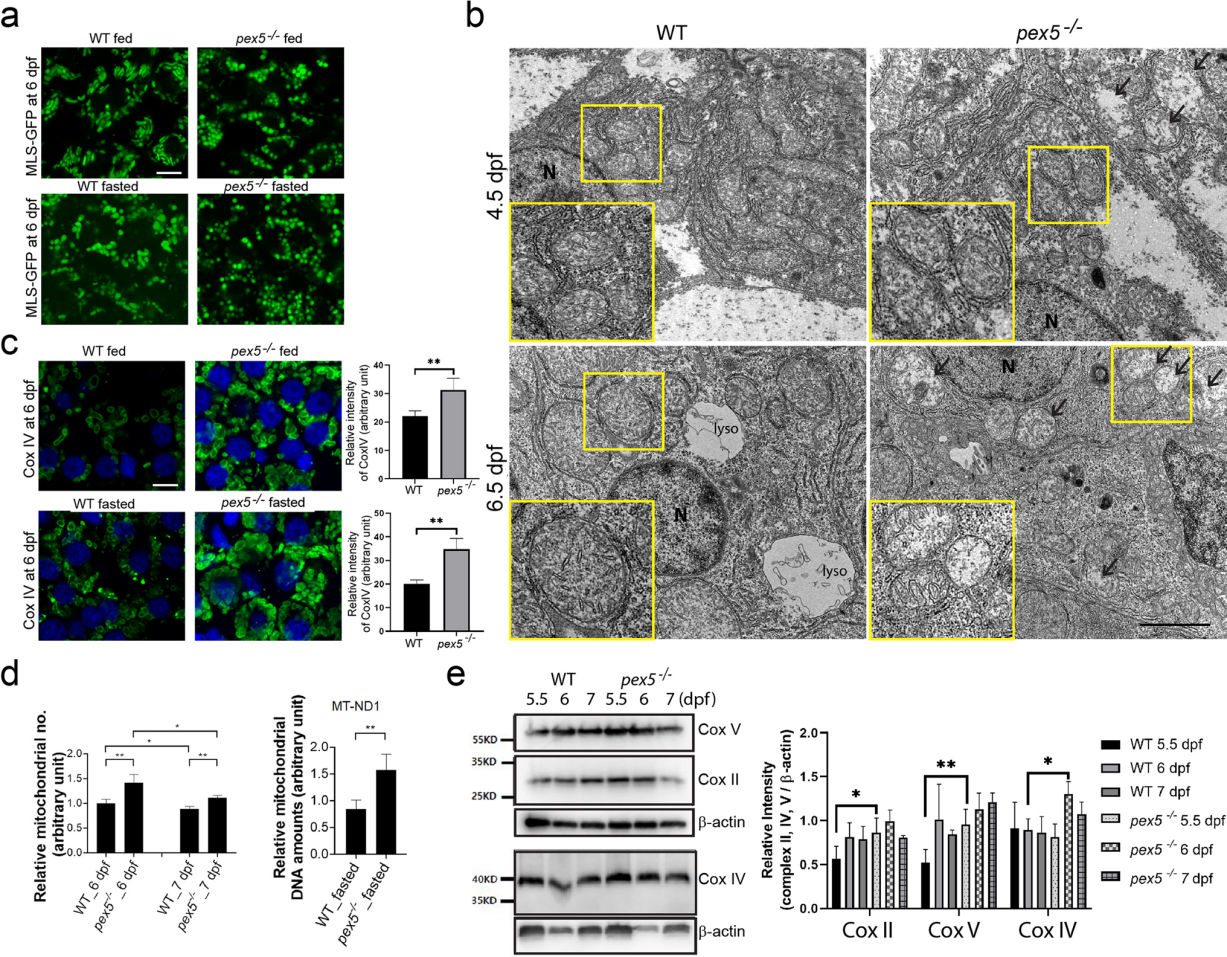
Fasting induces a radical change in the mitochondrial shape and abundance. **a** Expression of MLS-EGFP from the liver sections of *Xla.Eef1a1:MLS-EGFP* transgenic zebrafish at 6 dpf is shown to compare between the WT and *pex5*^*−/−*^ zebrafish and between the feeding and fasting conditions. Scale bar = 5 μm. **b** Transmission electron microscope (TEM) images are shown to detect the differential mitochondrial shapes in the liver at 4.5 dpf and 6.5 dpf upon fasting depending on the *pex5* genotype. Arrows indicate mitochondria with abnormal cristae structure. Scale bar = 500 nm. **c** Endogenous expression of a component of CoxIV is shown following immunofluorescence for comparison between the WT and *pex5*^−/−^ zebrafish liver and between the feeding and fasting conditions. Scale bar = 5 μm. Graphs show quantified signal intensity of CoxIV at the indicated conditions and presented as the average with error bars indicating standard deviation. Statistical significance was determined using the Student’s *t* test in Microsoft Excel; ** indicates *p* values < 0.01. **d** Mitochondrial abundance was analyzed by either counting the number of mitochondria in the liver cells of WT and *pex5*^*−/−*^ zebrafish at the indicated stages under fasting conditions (left) or quantifying the mitochondrial DNA amount at 6 dpf in reference to a β-actin genomic region using quantitative polymerase chain reaction (qPCR) (right). Note that the number of mitochondria in the liver cells of fasted WT zebrafish at 6 dpf was set to 1, which was used for reference to quantify those in the rest of the samples. Statistical significance was determined using the Student’s *t* test in Microsoft Excel; * and ** indicate *p* values < 0.05 and < 0.01, respectively. **e** Western blot using whole lysates was performed to compare expression levels of component proteins of the respiratory chain complexes, II V, and IV between WT and *pex5*^*−/−*^ at the indicated developmental stages. A representative image is shown, and the expression levels were quantified and presented as the average of three repeats with error bars indicating standard deviation. Statistical significance was determined using the Student’s *t* test in Microsoft Excel; * and ** indicate *p* values < 0.05 and < 0.01, respectively

Previous studies using knockout mice have shown that mitochondrial abnormalities could stem from dysfunctional peroxisomes [11, 17, 18, 36]. To evaluate the effect of the *pex5* mutation and nutritional stress on mitochondria, we first examined mitochondrial morphology using the expression of *Xla.Eef1a1:MLS-EGFP* transgene and compared it between WT and *pex5*^*−/−*^ under feeding and fasting conditions (Fig. 3a). Under feeding conditions, mitochondria were largely elongated in the hepatocytes of WT at 6 dpf (*n = *8), whereas they were mostly fragmented in *pex5*^−/−^ (*n = *8), suggesting that *pex5* deficiency might cause mitochondria to be morphologically different from those in WT under feeding conditions. Consistent with this idea, mitochondria in the hepatocytes of *pex5*^*−/−*^ were fragmented as early as 5 dpf (Supplementary Fig. S2, *n = *8). The different morphologies of mitochondria were maintained at 7 dpf under feeding conditions (Fig. 3a and Supplementary Fig. 2, *n = *8). These results indicate that mitochondria in WT hepatocytes under feeding conditions maintained their elongated morphology, while those in *pex5*^*−/−*^ underwent a morphological change to become largely fragmented.

Notably, when zebrafish were subjected to fasting, some mitochondria in the WT hepatocytes became fragmented and formed an elliptical shape at 6 dpf (Fig. 3a, *n* = 8), indicating that fasting might induce a change in mitochondrial shape. When the same fasting regime was imposed on *pex5*^−/−^, most mitochondria in the liver formed a round shape at 6 dpf and remained similar at 7 dpf (Supplementary Fig. 2, *n = *8), suggesting that fasting promoted further changes in mitochondrial shape in *pex5*^*−/−*^. To further examine the mitochondrial structural changes dependent on the *pex5* genotype and nutritional status, we examined liver sections using TEM. Consistent with the fluorescence microscopic observation, TEM showed that elongated mitochondria were observed in the WT liver, whereas mitochondria were fragmented in the *pex5*^*−/−*^ liver at 4.5 dpf (Fig. 3b). Moreover, unlike the mitochondria in the WT liver, few mitochondria appeared to be damaged in the *pex5*^*−/−*^ livers as their cristae structure was disrupted. Fasting facilitated mitochondrial fission, as mitochondria of WT liver at 6.5 dpf were no longer tubular. However, the mitochondria in the fasted WT liver were structurally intact and formed an elliptical shape. In contrast, in the livers of fasted *pex5*^*−/−*^, most mitochondria were found to be damaged and their cristae were disorganized at 6.5 dpf. These results suggest that mitochondria might adopt different shapes depending on the *pex5* genotype (i.e., WT vs. *pex5*^−/−^) and metabolic state (fed vs. fasted), thereby displaying the most severely deranged shape in the liver cells of *pex5*^*−/−*^ under fasting conditions.

Since different mitochondrial shapes can be translated into different qualities [43–45], we examined mitochondrial abundance under different conditions. Immunostaining showed significantly increased expression of the mitochondrial proteins belonging to respiratory chain complex IV in the fasted *pex5*^−/−^ hepatocytes compared to that in the fasted WT at 6 dpf (Fig. 3c, *n* = 8/genotype). In addition, we found that the number of mitochondria in the liver cells of fasted *pex5*^*−/−*^ was 1.4 times higher than that in fasted WT at 6 dpf (Fig. 3d). The increased mitochondrial abundance in *pex5*^*−/−*^ liver cells was correlated with increased mitochondrial DNA content, as determined by the amount of the *MT-ND1* gene, which encodes a component of the respiratory chain complex I (Fig. 3d). The expression of other mitochondrial genes, such as *MT-ATP6* and *MT-CYB*, also showed a similar trend (data not shown). Although we were unable to perform western blot analysis using the larval liver due to limited materials, results from western blotting using whole larval lysates showed increased levels of respiratory chain complex proteins in the fasted *pex5*^−/−^ at 5.5 - and 6 dpf depending on complex proteins compared to those in the fasted WT (Fig. 3e), further supporting elevated mitochondrial abundance in the fasted *pex5*^*−/−*^ animals.

To understand the molecular mechanism underlying the mitochondrial phenotype in fasted *pex5*^*−/−*^ livers, we performed RNA-seq analysis using total RNAs prepared from fasted WT and *pex5*^*−/−*^ livers at 6 dpf and compared gene expression profiles between the two. The gene expression pattern was not biased to either side and was evenly distributed, indicating that there were no technical artifacts that might affect the subsequent analysis (Supplementary Fig. S3a). We compared the expression profiles of mitochondrial genes, which were defined by MitoCarta3.0 [46], and OXPHOS genes between the two genotypes (Supplementary Fig. S3b, c). We also compared the expression of genes involved in mitochondrial biogenesis between the two (Supplementary Fig. S3d). By examining the fold changes in gene expression, we found that approximately 75% of OXPHOS genes and 59% of mitochondrial genes were upregulated in fasted *pex5*^−/−^ livers (Supplementary Fig. S3e). Moreover, eight out of ten genes involved in mitochondrial biogenesis were upregulated in fasted *pex5*^*−/−*^ livers compared to those in fasted WT livers. These results suggest that the fasted *pex5*^*−/−*^ livers, in comparison with the fasted WT livers, had greater mitochondrial quantity and possibly increased mitochondrial function.

### Fasting increases hepatic ROS and facilitates depletion of hepatic lipids and glycogen in *pex5*^−/−^

**Fig. 4:**
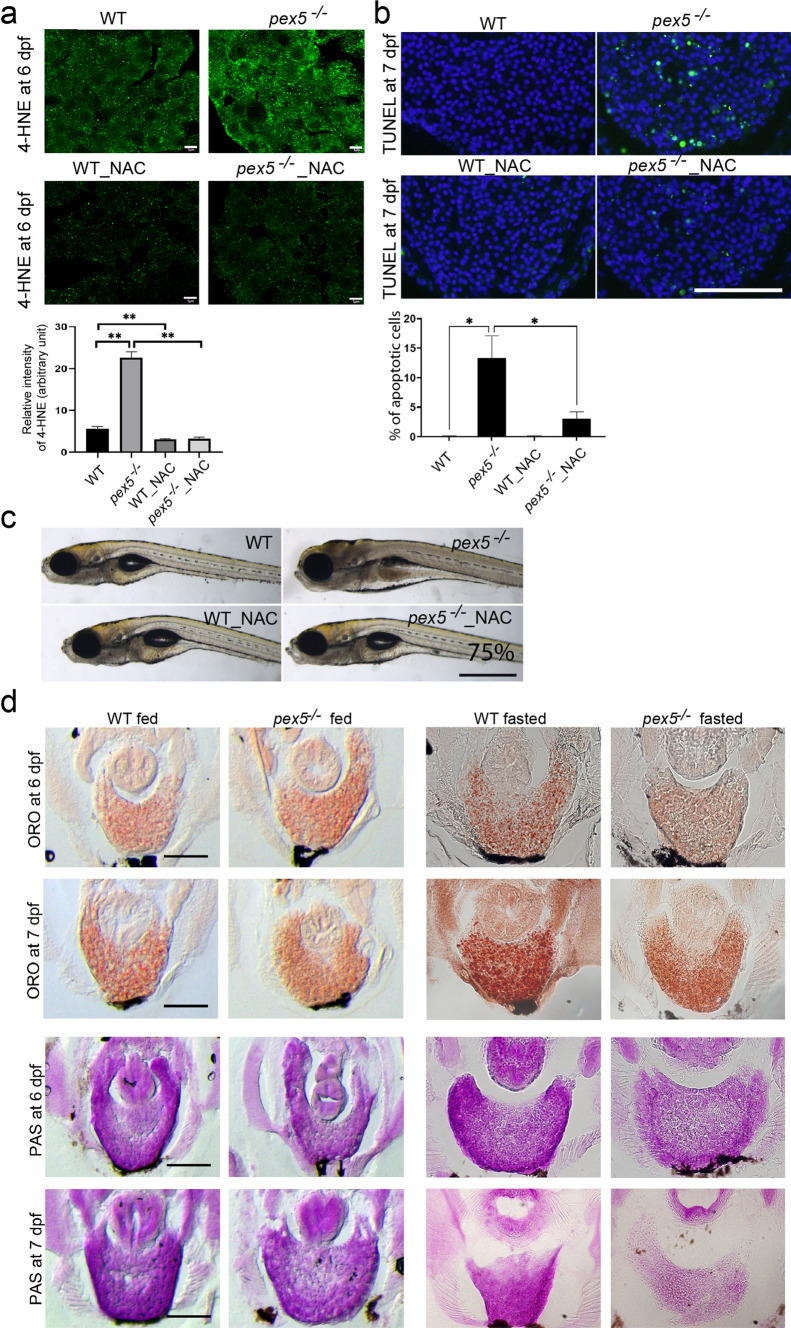
Fasting induces ROS and abnormally increased consumption of hepatic lipids and glycogen in the *pex5*^*−/−*^ liver. **a** 4-hydroxynonenal (4-HNE) was performed in the liver sections of WT and *pex5*^*−/−*^ zebrafish at 6 dpf either untreated or treated with 100 μM N-acetyl-cysteine (NAC). Scale bar = 5 μm. Graph shows quantified signal intensity and presented as the average with error bars indicating standard deviation. Statistical significance was determined using the Student’s *t* test in Microsoft Excel; ** indicates *p* values < 0.01. **b** Terminal deoxyribonucleotidyl transferase (TDT)-mediated dUTP nick-end labeling (TUNEL) staining was performed in the liver sections of WT and *pex5*^*−/−*^ zebrafish at 7 dpf either untreated or treated with NAC. Positive signals appear in green, and nuclei were stained with 4ʹ, 6-diamidino-2-phenylindole (DAPI) in blue. Scale bar = 100 μm. Graph shows the percentage of apoptotic cells per different conditions with error bars indicating standard deviation. Statistical significance was determined using the Student’s *t* test in Microsoft Excel; * indicates *p* values < 0.05. **c** Representative images of WT and *pex5*^*−/−*^ zebrafish at 9 dpf either untreated or treated with NAC. Larvae were shown in lateral views with anterior to the left. Scale bar = 1 mm. **d** ORO (upper panels) or PAS staining (lower panels) was performed in the liver sections of both fed and fasted WT and *pex5*^*−/−*^ zebrafish at 6 and 7 dpf. Representative images are shown. Scale bar = 100 μm

It is conceivable that elevated mitochondrial activity may induce high ROS levels. We tested whether this was the case with fasted *pex5*^*−/−*^ livers. We used 4-hydroxynonenal (4-HNE) level as a measure of ROS-dependent lipid peroxidation [47, 48]. *Pex5*^*−/−*^ liver cells showed a strong increase in the level of 4-HNE compared to WT liver cells during fasting at 6 dpf (Fig. 4a and b, *n* = 12). Consistent with ROS as a potent apoptotic inducer, we found a significant increase in apoptotic cell death in the liver of *pex5*^−/−^ at 7 dpf (Fig. 4c and d, *n* = 12). We attempted to relieve the ROS burden by treating with NAC, a well-known ROS scavenger, which dramatically reduced 4-HNE levels in the liver cells of all treated larvae (Fig. 4a, *n* = 12) and remarkably decreased the number of apoptotic liver cells of *pex5*^−/−^ compared to the untreated *pex5*^−/−^ (Fig. 4b, *n* = 12). Importantly, NAC treatment almost completely rescued 75% of fasted *pex5*^*−/−*^ from developing lethality-associated phenotypes (Fig. 4c, *n* = 24). These results suggest that ROS induction in the liver cells of *pex5*^−/−^ at 6 dpf might also contribute to the early lethality of *pex5*^*−/−*^ under fasting conditions.

Given the importance of mitochondria in regulating cellular metabolism, we examined whether the nutritional status of *pex5*^*−/−*^ livers would differ from that of WT livers upon fasting. We performed Oil Red O (ORO) and periodic acid-Schiff (PAS) staining of WT and *pex5*^*−/−*^ zebrafish to detect neutral lipids and glycogen, respectively. There was no noticeable difference between the two genotypes under feeding conditions (Fig. 4d, *n* = 6). However, fasting considerably reduced the levels of both neutral lipids and glycogen in *pex5*^*−/−*^ livers at 6 and 7 dpf (*n = *6), suggesting that fasting might induce metabolic disturbances in *pex5*^−/−^ zebrafish. As NAC treatment repressed apoptotic cell death and mortality in *pex5*^*−/−*^, we also examined the effect of NAC on liver metabolism. NAC treatment effectively restored the levels of both hepatic lipids and glycogen in fasted *pex5*^*−/−*^ compared to the untreated group at 6 dpf (Supplementary Fig. S4, 67%, *n = *6), indicating that ROS is a detrimental factor for metabolic disturbance in fasted *pex5*^*−/−*^.

### Limiting mitochondrial activity partially rescues the expedited death of fasted *pex5*^−/−^

**Fig. 5:**
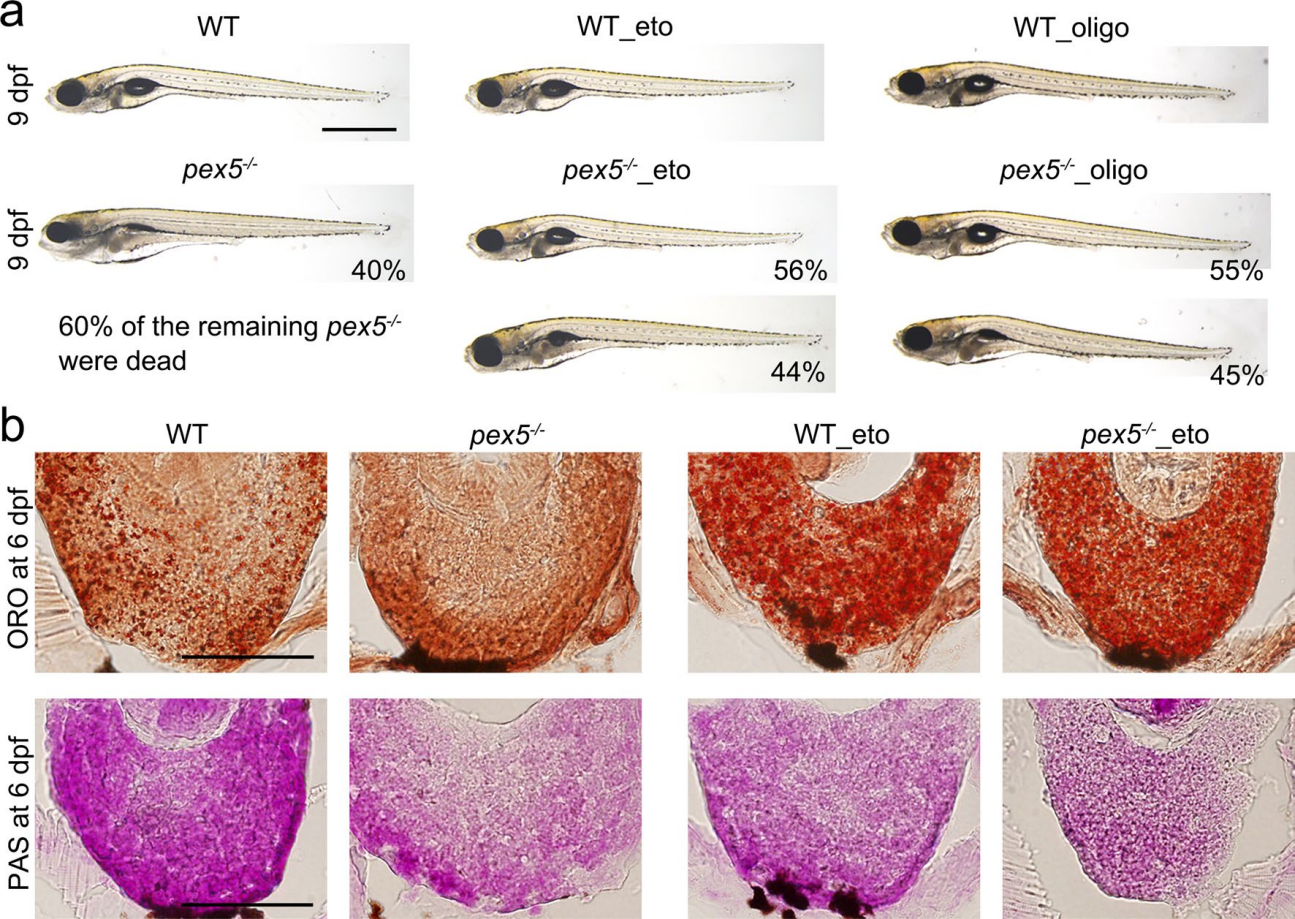
Inhibiting either the mitochondrial β-oxidation or ATP synthase ameliorates the fasting-associated *pex5*^*−/−*^ phenotypes. **a** Either etomoxir (1.5 μM) or oligomycin (5 pM) was treated from 5 to 9 dpf. A representative image from the survived 40% of the fasted, untreated *pex5*^*−/−*^ zebrafish is shown, and the remaining 60% larvae were found dead at 9 dpf as indicated. The average percentage representing the degree of rescue from two independent experiments is presented in each panel. Images were shown in lateral views with anterior to the left. Scale bar = 1 mm. **b** Liver sections from the WT and *pex5*^−/−^ zebrafish at 6 dpf treated with either etomoxir (eto, 1.5 μM) or oligomycin (oligo, 5 pM) from 5 dpf were shown following either ORO (upper panels) or PAS staining (lower panels). The same staining of liver sections from untreated WT and *pex5*^−/−^ zebrafish at 6 dpf was shown for comparison. A representative image for each condition is shown. Scale bar = 100 μm

Our observations of elevated mitochondrial activity, high 4-HNE levels, and depletion of hepatic lipids in fasted *pex5*^−/−^ livers led us to hypothesize that *pex5*^−/−^ could be sensitized to induce metabolic disturbances in the liver under fasting conditions. Therefore, we investigated whether partial inhibition of the mitochondrial pathway could rescue metabolic disturbances in fasted *pex5*^−/−^. Treatment with etomoxir, a well-known chemical inhibitor of carnitine palmitoyltransferase 1a necessary for fatty acid beta-oxidation, was able to rescue morphological defects in 56% (*n = *36) of fasted *pex5*^−/−^, all of which would otherwise develop fasting-induced phenotypes and associated early mortality at 9 dpf (Fig. 2c; Fig. 5a). A similar rescue (55%, *n = *22) was also observed following treatment with oligomycin A, an inhibitor of mitochondrial F_0_F_1_-ATPase, supporting our hypothesis that fasting may abnormally induce mitochondrial activity and that limiting mitochondrial function thus rescues the phenotypes of fasted *pex5*^−/−^. Consistent with mitochondrial abnormalities in the fasted *pex5*^*−/−*^, we found that treatment with etomoxir significantly reduced ROS levels in the *pex5*^*−/−*^ liver (Supplementary Fig. S5, *n = *5). We next tested whether the rescue effect observed with partial inhibition of mitochondrial function was mediated by ameliorating metabolic disturbances in fasted *pex5*^−/−^ livers. We found that both fasted WT and *pex5*^−/−^ treated with etomoxir accumulated hepatic lipids, as detected by ORO staining, compared to their untreated counterparts (Fig. 5b, *n* = 6/genotype), suggesting that hepatic lipid synthesis in fasted *pex5*^−/−^ might be functional, but hepatic lipid consumption was abnormally increased. Owing to the compromised fatty acid oxidation by etomoxir treatment, glycogen levels detected by PAS staining were accordingly decreased in the livers of both fasted WT and *pex5*^−/−^ treated with etomoxir compared to those in untreated counterparts (*n = *6/genotype). We also found that oligomycin treatment similarly accumulated hepatic lipids, but did not induce differences in glycogen content in the fasted *pex5*^*−/−*^ livers compared to those in the fasted WT livers (Supplementary Fig. S4, *n = *6). The differential effect on glycogen content between etomoxir and oligomycin might stem from the fact that etomoxir is specific to fatty acid oxidation. These results strongly suggest that fasted *pex5*^−/−^ larvae might have abnormally elevated mitochondrial activity, which could potentially be deleterious to the maintenance of metabolic balance and animal survival.

### *pex5* depletion increases mTORC1 activity under fasting conditions

**Fig. 6:**
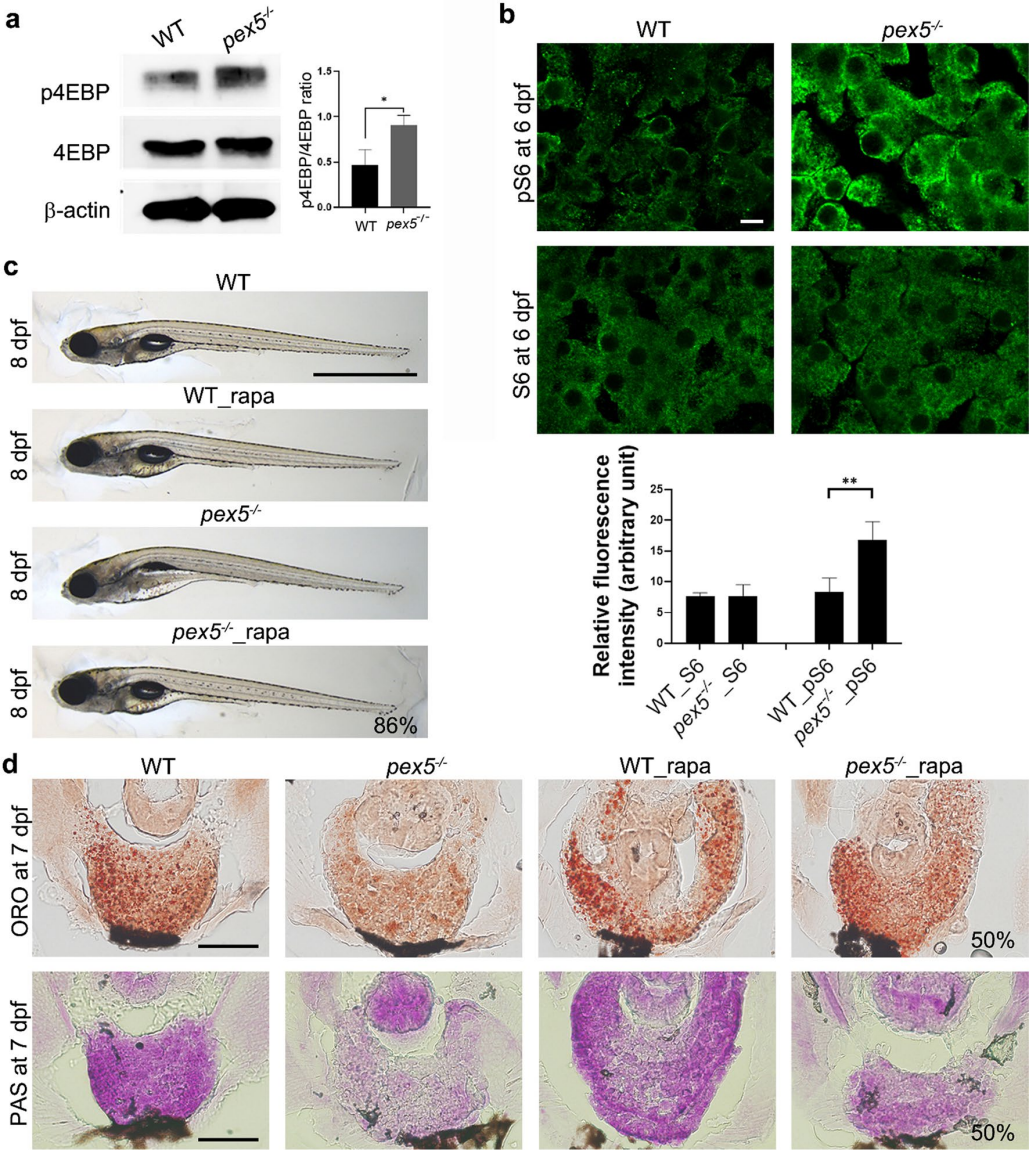
Mechanistic target of rapamycin complex 1 (mTORC1) signaling remains unrepressed in the fasted *pex5*^*−/−*^ liver. **a** Western blotting shows the expression levels of 4EBP and its phosphorylated form from whole lysates of WT and *pex5*^*−/−*^ zebrafish at 6 dpf. The expression of β-actin was used as the loading control. Graph shows quantified expression levels of p4EBP relative to β-actin levels and presented as the average of three repeats with error bars indicating standard deviation. Statistical significance was determined using the Student’s *t* test in Microsoft Excel; * indicates *p* value < 0.05. **b** Immunofluorescence was used to detect the differential levels of S6 and its phosphorylated form in the liver cells of WT and *pex5*^*−/−*^ at 6 dpf. DAPI detects nuclei in each liver section. Scale bar equals to 5 μm. Graph shows relative fluorescence intensity presented as the average with error bars indicating standard deviation. Statistical significance was determined using the Student’s *t* test in Microsoft Excel; ** indicates *p* values < 0.01. **c** Rapamycin (rapa, 250 nM) was used to treat WT and *pex5*^*−/−*^ from 3.5 to 8 dpf and representative images of the resulting larvae were laterally presented with anterior to the left. The average percentage representing a near-complete rescue from two independent experiments is indicated. Scale bar = 1 mm. **d** Liver sections from the larvae treated with rapamycin from 3.5 to 7 dpf were stained either ORO or PAS. Representative images are shown, and the percentage of rescue in the levels of liver lipids and glycogen are indicated in the panels of the fasted *pex5*^*−/−*^ zebrafish treated with rapamycin. Scale bar = 100 μm

A previous study using cell lines suggested that peroxisomes may act as signaling nodes to recruit tuberous sclerosis complex 2 (TSC2) in response to ROS, which then suppresses mammalian target of rapamycin complex 1 (mTORC1) activity [24]. In addition, we previously reported that PEX5 knockdown in HepG2 cells failed to suppress mTORC1 signaling in response to fasting [25]. Therefore, we tested whether fasting impairs mTORC1 signaling in *pex5*^−/−^. Western blotting using whole larval lysates for the expression of eukaryotic translation initiation factor 4E binding protein 1 (4E-BP1), a well-known mTORC1 downstream effector, indicated that the activated phosphorylated form of 4E-BP1 was increased in *pex5*^*−/−*^ under fasting conditions compared to that in WT (Fig. 6a). Moreover, the fasted *pex5*^−/−^ livers displayed highly increased phospho-S6, an activated form of S6 ribosomal protein, compared to the fasted WT hepatocytes (Fig. 6b, *n* = 8), supporting our observation of dysregulated mTORC1 signaling in the fasted *pex5*^−/−^.

To examine the association between dysregulated mTORC1 and larval defects in fasted *pex5*^*−/−*^ zebrafish, we used rapamycin to inhibit downstream effectors of mTORC1 [49]. Notably, rapamycin treatment efficiently rescued the morphological phenotypes observed in the fasted *pex5*^*−/−*^ (Fig. 6c, 86%, *n = *28), a phenomenon similar to the mitochondrial β-oxidation inhibition shown in Fig. 5. Moreover, rapamycin treatment effectively restored the levels of hepatic lipids of the fasted *pex5*^*−/−*^ much higher than the untreated group at 7 dpf (50%, *n = *8). Hepatic glycogen levels in fasted *pex5*^*−/−*^ zebrafish also increased upon rapamycin treatment (Fig. 6d, 50%, *n = *8). These results confirm that sustained mTORC1 activity during fasting might play an important role in the mortality of *pex5*^*−/−*^.

Given that both deregulated mitochondria and unrepressed mTORC1 are major contributors to larval defects upon fasting in *pex5*^*−/−*^ zebrafish, we determined the relationship between these two pathways. The role of mTORC1 in regulating mitochondrial biogenesis and function is well established [50, 51]. Indeed, rapamycin treatment restored mitochondrial abnormalities in fasted *pex5*^*−/−*^ livers, as evidenced by the reduced levels of both mitochondrial abundance and 4-HNE (Supplementary Fig. S5 and S6, *n = *5). We next examined the possibility that modulating mitochondrial function could, in turn, affect mTORC1 activity. Indeed, a highly enriched signal from anti-phospho-S6 immunofluorescence in fasted *pex5*^*−/−*^ livers at 6 dpf, indicative of dysregulated mTORC1 activity, was considerably decreased after etomoxir treatment (Supplementary Fig. S7, *n = *5). Accordingly, we tested the combinatorial role of mTORC1 and mitochondrial activities in contributing to phenotype severity and mortality in fasted *pex5*^*−/−*^ zebrafish by assessing morphological rescue when both were simultaneously inhibited. Full doses of rapamycin (250 nM) and etomoxir (1.5 μM) in combination resulted in developmental abnormalities in both WT and *pex5*^*−/−*^ zebrafish, presumably because of insufficient signaling required for normal larval development (*n = *15, not shown). However, a combination of a quarter dose of rapamycin and etomoxir rescued 53% of the fasted *pex5*^*−/−*^ (*n = *34). A quarter dose of each drug did not improve the phenotype observed in fasted *pex5*^*−/−*^ (*n = *12). These results indicate the presence of a positive relationship between mitochondrial function and mTORC1 activity in promoting larval defects in *pex5*^*−/−*^.

### Autophagy flux is impaired and damaged mitochondria are accumulated in *pex5*^−/−^ under fasting conditions

**Fig. 7:**
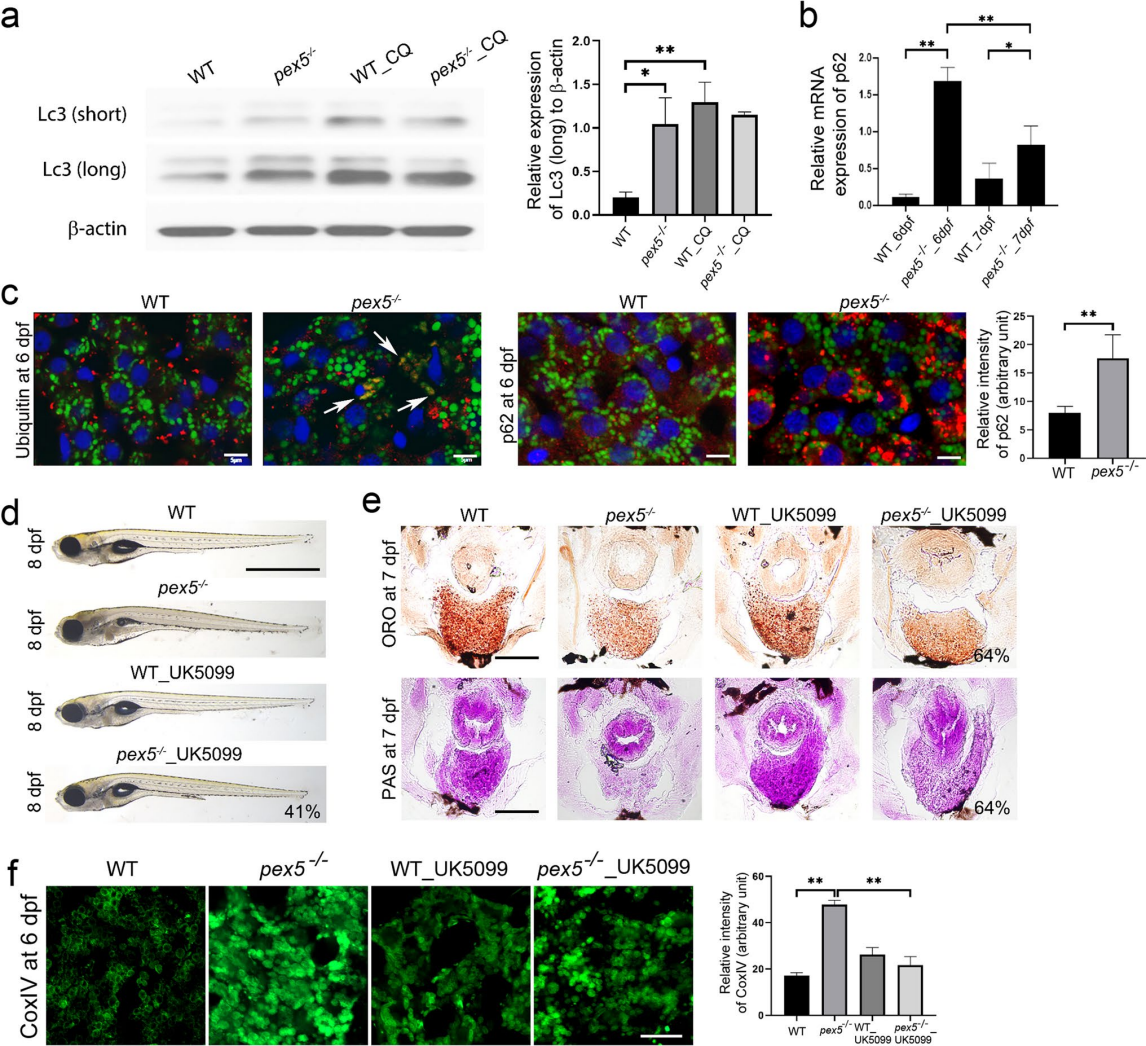
Dysfunctional mitochondria are accumulated in the fasted *pex5*^*−/−*^ liver. **a** Western blotting shows the level of the light chain 3 (Lc3)-II from whole lysates of WT and *pex5*^*−/−*^ at 6 dpf with or without chloroquine (CQ, 250 μM). Short and long stand for shorter and longer exposure for film development, respectively. The expression of β-actin was used as the loading control. Graph shows quantified expression levels of Lc3-II (long) relative to β -actin levels and presented as the average of three repeats with error bars indicating standard deviation. Statistical significance was determined using the Student’s *t* test in Microsoft Excel; * and ** indicate *p* values < 0.05 and < 0.01, respectively. **b** Relative expression levels of *p62* were determined in reference to those of β-*actin* by performing quantitative reverse transcription-polymerase chain reaction (qRT-PCR) using total RNA isolated from the liver cells of fasted WT and *pex5*^*−/−*^ zebrafish at the indicated stages. Data are presented as the average of three repeats for the relative ratio of p62/*b-actin* expression. Error bars indicate the standard deviation. Statistical significance was determined using the Student’s *t* test in Microsoft Excel; * and ** indicate *p* values < 0.05 and < 0.01, respectively. **c** Immunofluorescence was performed in liver sections from WT and *pex5*^*−/−*^ to detect damaged mitochondria using an anti-Ubiquitin antibody or an autophagy cargo protein using anti-p62 antibody in red as indicated. Mitochondria were visualized through the EGFP signal expressed in the transgenic *Tg*(*Xla.Eef1*α*1:MLS-EGFP*) zebrafish. DAPI stains nuclei in blue in each liver section. Representative images were shown from two independent experiments. Scale bar = 5 μm. Graphs show quantified signal intensity of p62 and presented as the average with error bars indicating standard deviation. Statistical significance was determined using the Student’s *t* test in Microsoft Excel; ** indicates p value < 0.01. **d** UK5099 (25 μM) was treated in WT and *pex5*^*−/−*^ from 3.5 to 8 dpf, and representative images of the resulting larvae at 8 dpf were laterally presented with anterior to the left. The average percentage representing a near-complete rescue from two independent experiments is indicated. Scale bar equals 1 μm. **e** Liver sections from the larvae treated with UK5099 from 3.5 to 7 pf were stained either ORO or PAS. Representative images are shown and the percentage of rescue in the levels of liver lipids and glycogen is indicated in the panels of the fasted *pex5*^−/−^ treated with UK5099. Scale bar = 100 μm. **f** UK5099 (25 μM) was treated in WT and *pex5*^*−/−*^ from 3.5 to 6 dpf, and immunofluorescence was performed in liver sections using anti-CoxIV antibody. Representative images are shown. Scale bar = 5 μm. Graphs show quantified signal intensity of CoxIV at the indicated conditions and presented as the average with error bars indicating standard deviation. Statistical significance was determined using the Student’s *t* test in Microsoft Excel; ** indicates p value < 0.01

Activated mTORC1 is reportedly associated with the suppression of autophagy [52–56], although autophagy can be regulated independently of mTORC1 under some conditions [57–60]. In addition, developing embryos require a certain level of mTORC1 to ensure the proper anabolic signals necessary for animal development. We analyzed the autophagy marker light chain 3 (Lc3)-II, a lipidated form of Lc3, and found it to be increased in *pex5*^*−/−*^ compared to that in WT under fasting conditions (Fig. 7a). We tested whether the increased Lc3-II levels were due to enhanced autophagy or autophagy blockade by treatment with chloroquine (CQ), an autophagy blocker. CQ treatment induced Lc3-II levels in WT, indicating that CQ effectively blocked autophagic flux. However, the Lc3-II levels between untreated and CQ-treated *pex5*^*−/−*^ showed minimal changes, suggesting autophagy blockade in fasted *pex5*^*−/−*^. To verify this, we examined p62 levels, which are known to be regulated in accordance with autophagy activity [61]. We found strikingly high levels of both *p62* mRNA and p62 protein in the liver cells of fasted *pex5*^*−/−*^ (Fig. 7b, c), consistent with autophagy blockade in fasted *pex5*^*−/−*^. We next examined the quality control of mitochondria because autophagy blockade may also influence mitochondrial health, related to our finding of increased mitochondrial activity and abundance with altered morphology in the liver cells of *pex5*^*−/−*^ (Figs. 3 and 5). The ubiquitination of mitochondrial proteins may act as a signal for the selective autophagic degradation of damaged mitochondria, known as mitophagy [61]. We found that ubiquitinated proteins significantly accumulated in the mitochondria of the liver cells of fasted *pex5*^*−/−*^ compared to WT (Fig. 7c, n = 6), indicating accumulated mitochondrial damage.

We reasoned that increasing autophagy might relieve the limited autophagy and rescue the phenotypes in fasted *pex5*^*−/−*^. UK5099, an inhibitor of the mitochondrial pyruvate carrier, has been reported to decrease intracellular acetyl coenzyme A levels and thus induce autophagy in an mTOR-independent manner [62]. We found that treatment with UK5099 rescued 41% of the fasted *pex5*^*−/−*^ almost completely (*n = *32) and the remaining 59%, at least partially, based on morphological phenotypes at 8 dpf (Fig. 7d). Moreover, treatment with UK5099 restored hepatic lipid and glycogen contents, at least partially, in 64% of fasted *pex5*^*−/−*^ larvae at 7 dpf (Fig. 7e, n = 11). Notably, consistent with mitophagy blockade in fasted *pex5*^*−/−*^ livers, UK5099 treatment significantly reduced the expression of Complex IV proteins in fasted *pex5*^*−/−*^ livers (Fig. 7f, n = 6). These results suggest that reduced autophagy flux might contribute to the deterioration of fasted *pex5*^*−/−*^.

## Discussion

Zebrafish is a well-established vertebrate animal model that offers a combination of genetic and experimental advantages in addition to other animal model systems [42, 63], but its use has been limited in peroxisomal research. Nonetheless, a pioneering study revealed that zebrafish peroxisomes were the most abundant in the liver and renal tubular epithelium, with a distribution similar to that observed in mammals [26]. In addition, we and other researchers have recently shown that functional peroxisomes are essential for normal zebrafish embryogenesis [27, 28, 64]. In this study, we generated *pex5*^*−/−*^ and verified its utility as a zebrafish model of ZSD by providing evidence of phenocopy in a mouse model of peroxisome dysfunction and in human patients. An intriguing feature of *pex5*^−/−^ is an increased sensitivity to fasting, which leads to metabolic failure and expedited death.

It is generally accepted that in response to peroxisome deficiency, mitochondria may eventually become dysfunctional. For example, *PEX5*^−/−^ hepatocytes display functional and morphological anomalies in mitochondria, which are often accompanied by increased oxidative stress [65]. Therefore, accumulation of dysfunctional mitochondria, together with failure in metabolic regulation, might cause phenotypic presentation in peroxisome-deficient diseases. In this study, we showed that mitochondria in zebrafish liver cells adopted their shape depending on both the *pex5* genotype and the nutritional state. For example, under feeding conditions, the mitochondria in WT liver cells were largely elongated, whereas those in *pex5*^*−/−*^ were fragmented (Fig. 3a and Supplementary Fig. S2). Fasting facilitated fission to induce mitochondria with an elliptical shape in WT, but further induced them to constitute a round-shaped population in *pex5*^−/−^. TEM images were consistent with disorganized mitochondria in the liver cells of fasted *pex5*^*−/−*^ (Fig. 3b). In particular, under fasting conditions, liver cells of fasted *pex5*^−/−^ contained severely damaged mitochondria with distorted cristae and a disorganized membrane structure at 6.5 dpf. Further efforts are necessary to determine the causal relationship between mitochondrial morphology and activity under different nutritional conditions.

Our finding of severely decreased lipid and glycogen levels in the liver cells of fasted *pex5*^−/−^ zebrafish (Fig. 4) prompted us to examine the role of the mitochondria upon fasting. Previous studies have reported that the absence of functional peroxisomes perturbs carbohydrate metabolism and reduces sensitivity to low sugar environments [15, 19, 66]. Similarly, we also found near-complete depletion of liver glycogen in fasted *pex5*^−/−^ zebrafish. We attempted to rescue the expedited mortality of fasted *pex5*^−/−^ by supplementing with glucose, but did not succeed (data not shown). Notably, we found that blocking mitochondrial β-oxidation was efficient for the survival of fasted *pex5*^−/−^ zebrafish. A similar rescue in mortality was observed with an inhibitor of ATP synthase, suggesting that mitochondrial activity increased as an initial response to peroxisomal dysfunction. Indeed, several studies have demonstrated that, in response to peroxisomal dysfunction, mitochondrial activities were increased as a compensatory mechanism; mitochondrial matrix enzymes were more active in *Pex5*^−/−^ hepatocytes than in WT [17, 36], and mitochondria-dependent β-oxidation was increased despite the evident hepatosteatosis [18, 67]. In addition, *Pex5*^−/−^ hepatocytes produced slightly more ATP than control hepatocytes [11]. Due to technical restrictions in using zebrafish larval livers, we were unable to measure the mitochondrial oxygen consumption rate and lipid oxidation. However, we found an increase in the number of mitochondria, the amount of mitochondrial DNA, the expression levels of mitochondrial proteins (Fig. 3), and the expression of OXPHOS and mitochondrial genes, as well as genes involved in mitochondrial biogenesis in the fasted *pex5*^−/−^ (Supplementary Fig. S3), all of which suggested increased mitochondrial activity. Although the exact underlying mechanism needs to be further elucidated, collective transcriptional activation of the mitochondrial genes (Supplementary Fig. S3) might contribute to the elevated mitochondrial activity in *pex5*^−/−^ under fasting conditions. In support of increased mitochondrial activity, we found depleted lipid content and increased levels of cellular ROS in hepatocytes of fasted *pex5*^−/−^. We confirmed that ROS might also contribute to the observed mortality, as NAC treatment inhibited apoptosis in fasted *pex5*^−/−^ livers, restored hepatic lipids and glycogen levels, and suppressed early lethality in *pex5*^*−/−*^ (Fig. 4). While hepatic lipids seem to be critical for the survival of fasted *pex5*^−/−^, the level of carbohydrates may also be important. The relative importance of carbohydrate and lipid levels during zebrafish development is not well understood.

As a critical factor that influences the phenotypic severity of fasted *pex5*^−/−^, we identified mTORC1 as not being repressed under fasting conditions. mTORC1 facilitates anabolic reactions under nutrient-rich conditions but is repressed under energy-deprived conditions [52–56]. The role of peroxisomes in regulating mTORC1 signaling has been reported in a cell line in which peroxisomes recruit TSC2 to repress mTORC1 in response to ROS [24]. We also reported that the mTORC1 regulatory pathway is dependent on PEX5 upon serum starvation in HepG2 cells [25]. In this study, we confirmed increased mTORC1 activity under fasting conditions in the liver cells of *pex5*^−/−^ (Fig. 6). Persistent activation of mTORC1 increases the expression of genes involved in glycolysis [66] which likely explains the depleted hepatic glycogen levels in fasted *pex5*^−/−^. In addition**,** persistent mTORC1 activity likely regulates mitochondrial activity [50, 51, 68], thereby further enhancing mitochondrial activity in liver cells of fasted *pex5*^−/−^. Notably, we showed that mTORC1 was also affected by mitochondrial function. Etomoxir treatment remarkably decreased phospho-S6 levels in the liver cells of fasted *pex5*^−/−^ zebrafish (Supplemental Fig. S7), suggesting the presence of an axis that influences mTORC1 activity via the mitochondria. Furthermore, autophagy also influences the severity of the phenotype observed in fasted *pex5*^−/−^. Fasting may burden mitochondria-dependent energy production, leading to the accumulation of damaged mitochondria, which are not efficiently cleared due to limited autophagy flux (Fig. 7). Accordingly, an autophagy inducer was able to partially rescue the phenotypes of fasted *pex5*^*−/−*^, indicating that blockade of autophagy might be detrimental to the survival of fasted *pex5*^−/−^.

**Fig. 8:**
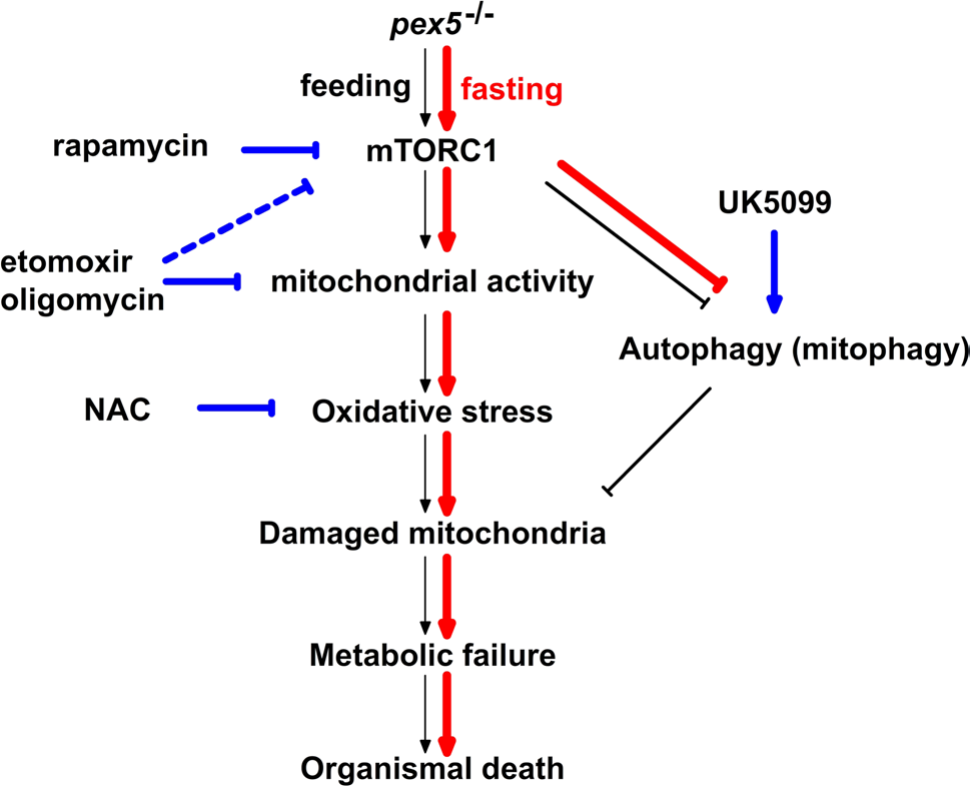
A model to illustrate the fasting-induced early mortality in *pex5*^*−/−*^ in this study. Events occurring primarily in the *pex5*^*−/−*^ liver under normal fed conditions are shown in black arrows. Briefly, due to dysfunctional peroxisomes, metabolic burden in *pex5*^*−/−*^ induces compensatory activation of mitochondria which may then lead to mitochondrial damage and subsequent metabolic failure and organismal death. The entire process may run slow and take almost one month presumably due to the availability of nutrients under fed conditions. However, the process becomes highly facilitated under fasting conditions. mTORC1 becomes repressed in the fasted WT liver but not in the *pex5*^*−/−*^ liver. In the fasted *pex5*^*−/−*^ liver, mitochondrial activities are upregulated by unrepressed mTORC1 whose activity may also regulated by mitochondrial activity. Further, abnormally high mitochondrial activities may rapidly induce mitochondrial dysfunction and lead to accumulation of damaged mitochondria, which may not be replenished due to the limited autophagic flux. The undesirable condition upon fasting then leads to metabolic failure and results in early mortality of *pex5*^*−/−*^. The events strengthened upon fasting are shown by red arrows. The points of action of drugs that either activate or inhibit the process are shown in blue

Based on the present study and previous findings, we propose a working model in which the phenotypes displayed in *pex5* deficiency become exacerbated upon fasting (Fig. 8). Peroxisome deficiency induces mitochondrial abnormalities and the associated metabolic failure. When fasting is imposed as a metabolic stress additive to the absence of functional peroxisomes, the entire pathogenesis seems to be greatly facilitated, resulting in rapid depletion of hepatic lipids and glycogens and, consequently, expedited organismal death. A critical player upon fasting would be unrepressed mTORC1 activity, by which mitochondrial function induced initially in response to peroxisome dysfunction could be further increased. Increased mitochondrial function may, in turn, prevent mTORC1 repression. This positive regulatory loop builds damaged mitochondria, which may not be efficiently cleared due to impaired autophagy flux in fasted *pex5*^*−/−*^ livers. Further studies to elucidate the factors that block autophagy flux upon fasting in the absence of functional peroxisomes will warrant a refinement in interpreting a series of molecular events in *pex5* deficiency under environmental stress.

Taken together, we showed that zebrafish can be a useful animal model system to provide mechanistic insights into peroxisome-dependent mitochondrial health. In addition, zebrafish may provide an in vivo experimental approach to reveal the metabolic response to environmental stresses, such as fasting. Based on the intervention experiments, we suggest that targeting mitochondrial function or mTORC1 individually or in combination may have a beneficial effect on the prevention of phenotypic exacerbation caused by peroxisome dysfunction during metabolic crisis. In parallel, we suggest that a drug that can reduce ROS or induce mitophagy may provide a preventive or therapeutic option for the treatment of peroxisomal dysfunction exhibiting metabolic imbalance.

## Supplementary Information

Below is the link to the electronic supplementary material.

Supplementary file1 (DOCX 3290 KB)

## Data Availability

All data generated or analyzed during this study are included in this published article and its supplementary information files.
