# Supplementary material for: Loss of pex5 sensitizes zebrafish to fasting due to deregulated mitochondria, mTOR, and autophagy
Source: Cell Mol Life Sci. 2023 Feb 23;80(3):69. doi: 10.1007/s00018-023-04700-3 (PMC9950184; doi:10.1007/s00018-023-04700-3)
Supplement: Supplementary file 1 — Supplementary file1 (DOCX 3290 KB) [file 18_2023_4700_MOESM1_ESM.docx]

**Supplementary Materials**

**Loss of *pex5* sensitizes zebrafish to fasting due to deregulated mitochondria, mTOR, and autophagy**

Sushil Bhandari^1,†^, Yong-Il Kim^1,†^, In-Koo Nam^2,3,†^, KwangHeum Hong^1,2^, Yunju Jo^2,4^, Kyeong-Won Yoo^5^, Weifang Liao^1,2^, Jae-Young Lim^1^, Seong-Jin Kim^2,6^, Jae-Young Um^7^, Peter K. Kim^8^, Ho Sub Lee^9^, Dongryeol Ryu^2,4^, Seok-Hyung Kim^2^, SeongAe Kwak^9,#^, Raekil Park^10,#^ and Seong-Kyu Choe^1,2,5,6,11,#^

^1^Department of Medicine, Graduate School, Wonkwang University, Iksan, 54538, South Korea

^2^Sarcopenia Total Solution Center, Wonkwang University, Iksan, 54538, South Korea

^3^Institute of Brain Science, Wonkwang University, Iksan, 54538, South Korea

^4^Department of Molecular Cell Biology, Sungkyunkwan University School of Medicine, Suwon 16419, South Korea

^5^Department of Microbiology, Wonkwang University School of Medicine, Iksan, 54538, South Korea,

^6^Department of Biomedical Science, Graduate School, Wonkwang University, Iksan, 54538, South Korea

^7^Department of Pharmacology, College of Korean Medicine, Kyung Hee University, Seoul, 02447, South Korea.

^8^Department of Biochemistry, University of Toronto, Toronto, ON, M5S 1A8, Canada

^9^Hanbang Cardio-Renal Research Center, Wonkwang University, Iksan, 54538, South Korea

^10^Department of Biomedical Science & Engineering, Institute of Integrated Technology, Gwangju Institute of Science & Technology, Gwangju, 61005, South Korea

^11^Institute of Wonkwang Medical Science, Wonkwang University, Iksan, 54538, South Korea

^†^ These authors contributed equally as first authors.


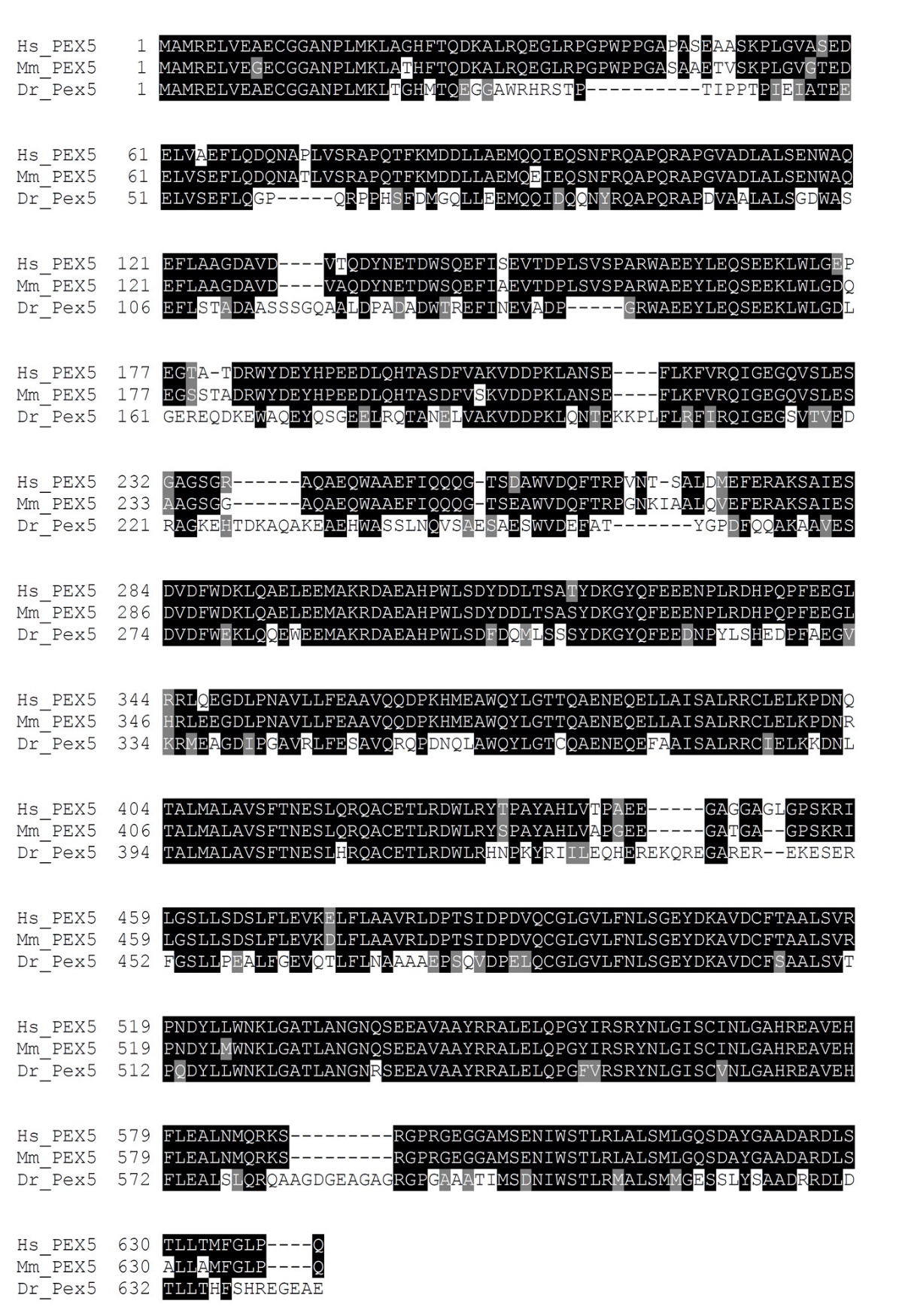


**Supplementary Fig. S1** Sequence alignment of the peroxin 5 (PEX5) proteins in humans, mice, and zebrafish. Identical amino acids are shaded in black boxes and similar sequences are shown in grey boxes. There is 96.3% sequence similarity between humans and mice and 71.3% between humans and zebrafish.


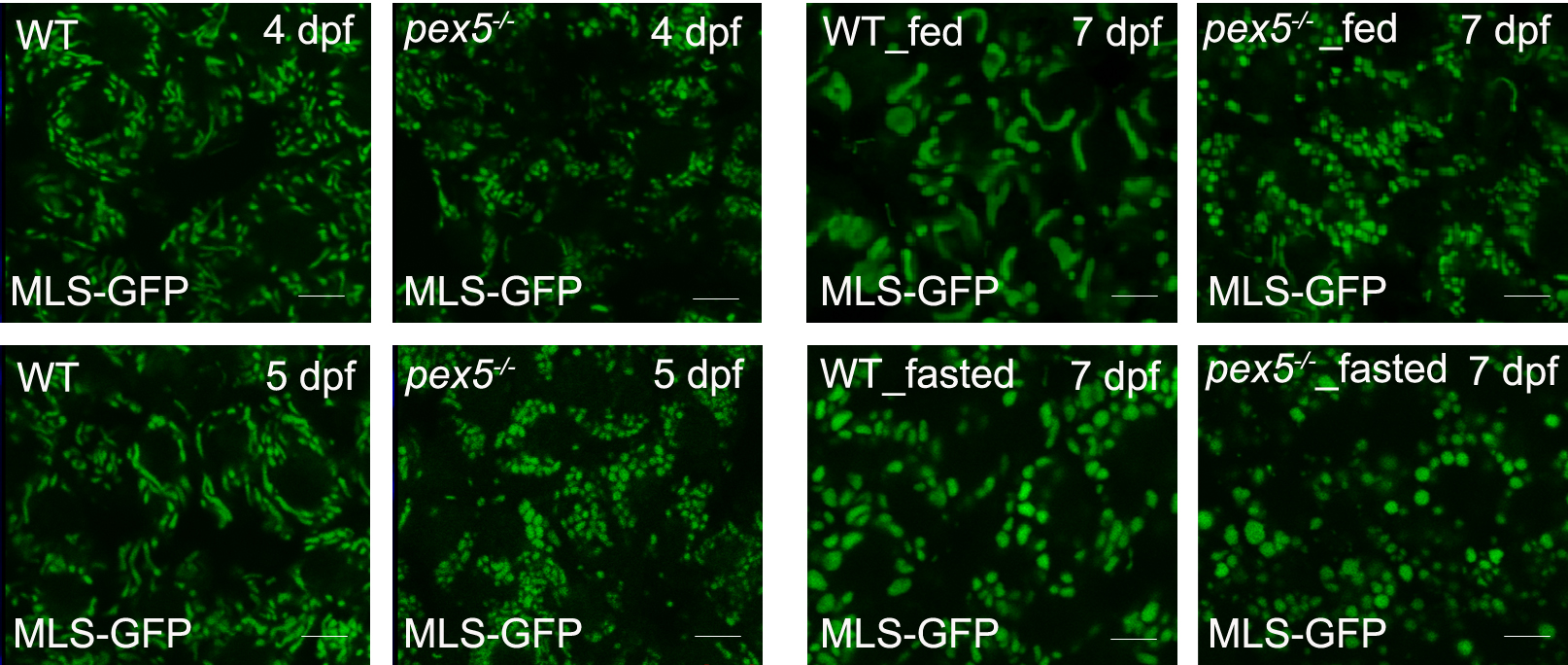


**Supplementary Fig. S2** Mitochondria adopt different morphologies in the liver cells of *pex5^-/-^* zebrafish compared to wild-type (WT). Confocal microscopy of MLS-enhanced green fluorescent protein (EGFP) from the *EF1α:MLS-EGFP* transgenic zebrafish was used to analyze the mitochondrial morphology in the liver sections of WT and *pex5^-/-^* zebrafish at the indicated developmental stages. Fed or fasted liver sections from WT and *pex5^-/-^* zebrafish at 7 dpf are indicated. Scale bar = 5 μm.


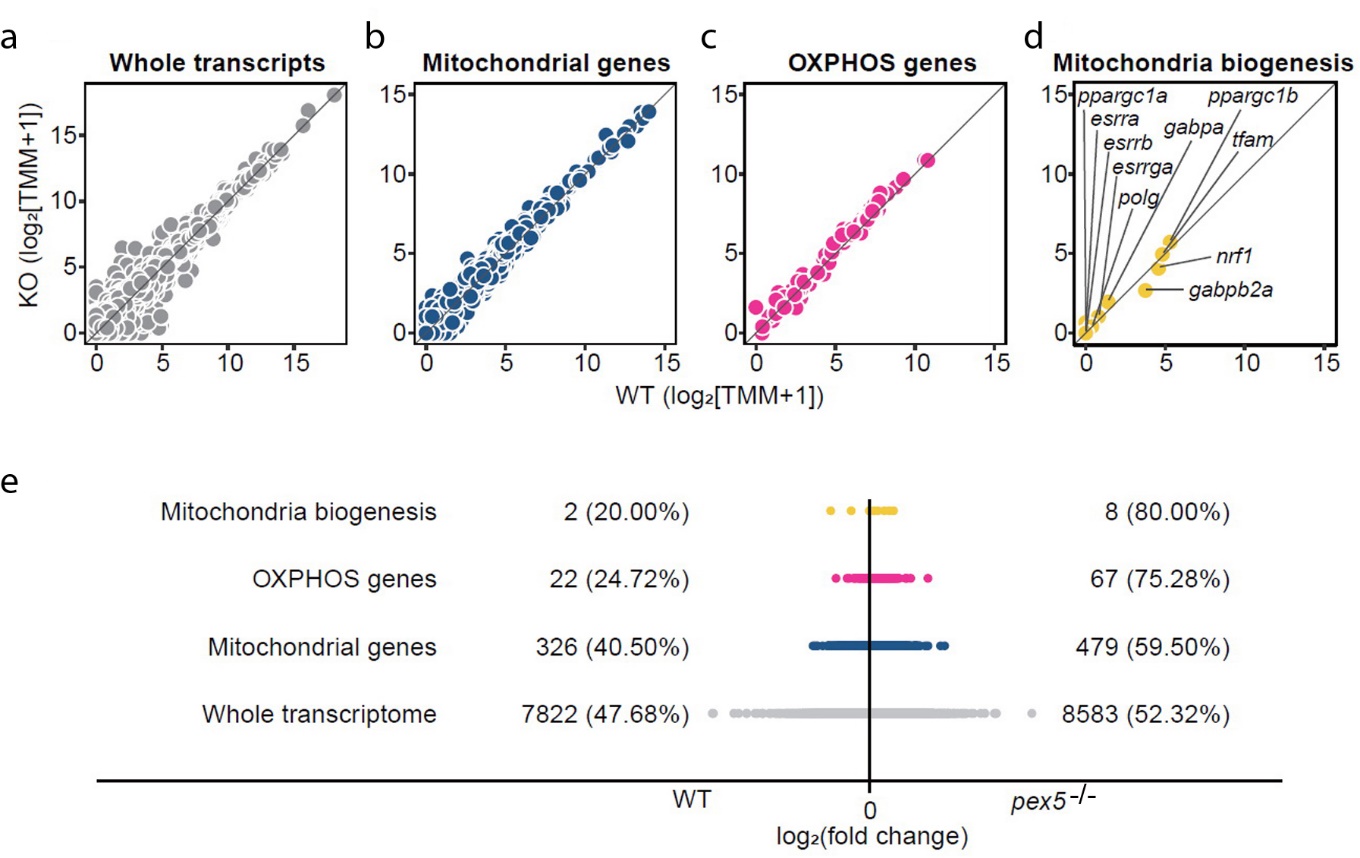


**Supplementary Fig. S3** Transcriptomic of WT and *pex5*^-/-^ zebrafish. (A-D) Scatter plots showing the gene expression profiles (log2[TMM+1]) of whole transcriptome (A), mitochondrial genes (B), OXPHOS genes (C) and mitochondrial biogenesis (D) in WT and *pex5^-/-^*. E. Scatter plots presenting differential gene-expression (log_2_[fold change]) of individual genes included in each indicated gene set (mitochondrial biogenesis [top], OXPHOS [second], mitochondrial genes [third], and whole transcriptome [bottom]) in WT and *pex5^-/-^* indicate that 75.28% of OXPHOS genes and 59.5% of mitochondrial genes were elevated in *pex5*^-/-^.


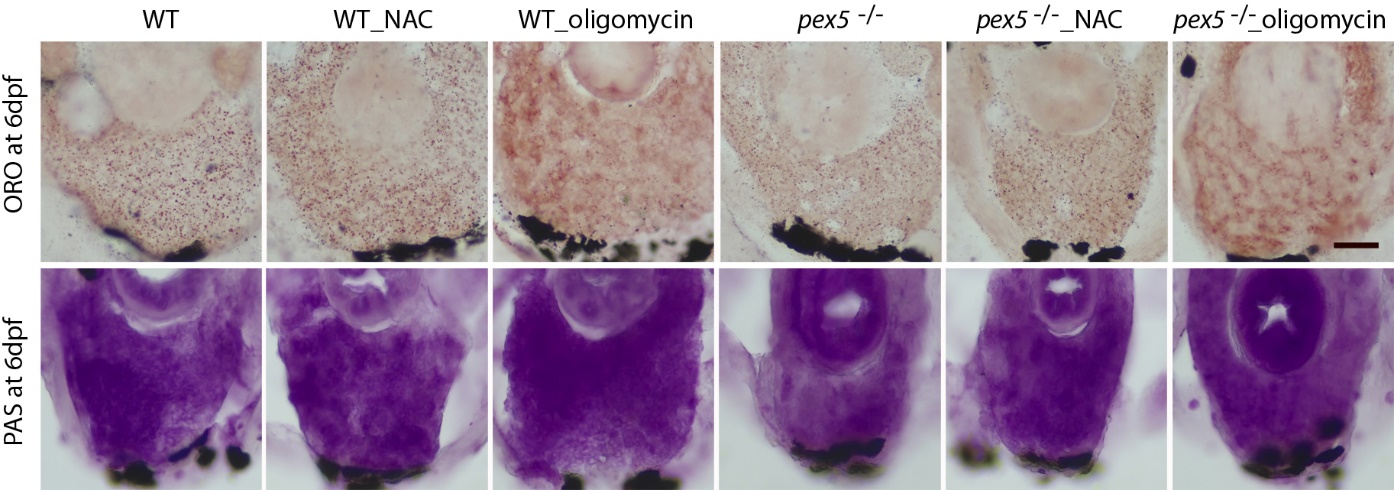


**Supplementary Fig. S4** Changes in metabolic features of fasted WT or *pex5*^-/-^ liver at 6 dpf. Liver sections from WT and *pex5*^-/-^ zebrafish at 6 dpf treated with either NAC or oligomycin (oligo, 5 pM) were shown following either ORO (upper panels) or PAS staining (lower panels). A representative image for each condition is shown. Scale bar = 50 μm


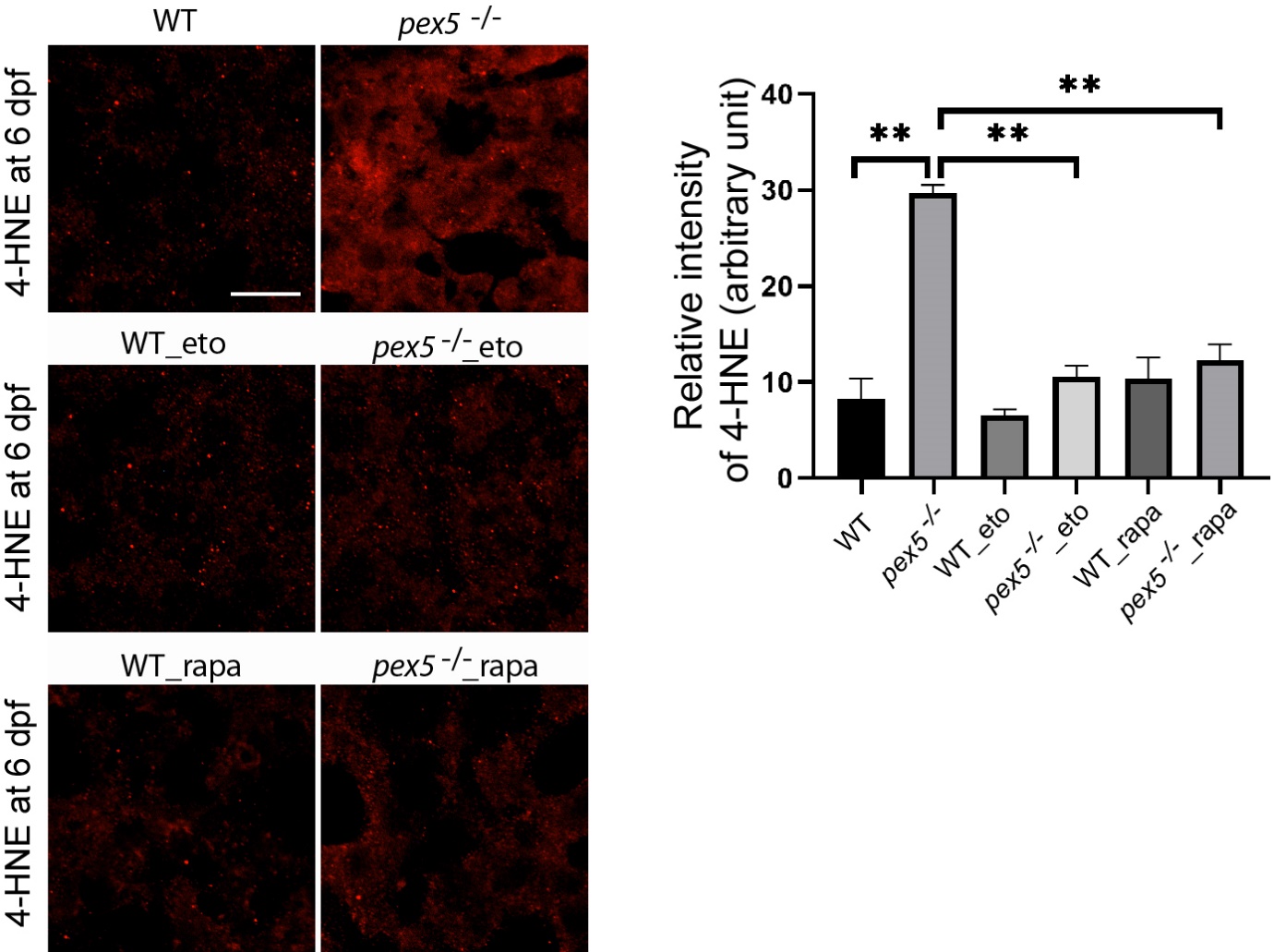


**Supplementary Fig. S5** Changes in ROS levels in the livers of WT or *pex5*^-/-^ treated with an inhibitor of either mitochondrial β-oxidation or mTOR. 4-hydroxynonenal (4-HNE) was performed in the liver sections of WT and *pex5^-/-^* zebrafish at 6 dpf either untreated or treated with etomoxir (eto) rapamycin (rapa). Scale bar = 10 μm. Graph shows quantified signal intensity and presented as the average with error bars indicating standard deviation. Statistical significance was determined using the Student t-test in Microsoft Excel; ** indicates p-values < 0.01.


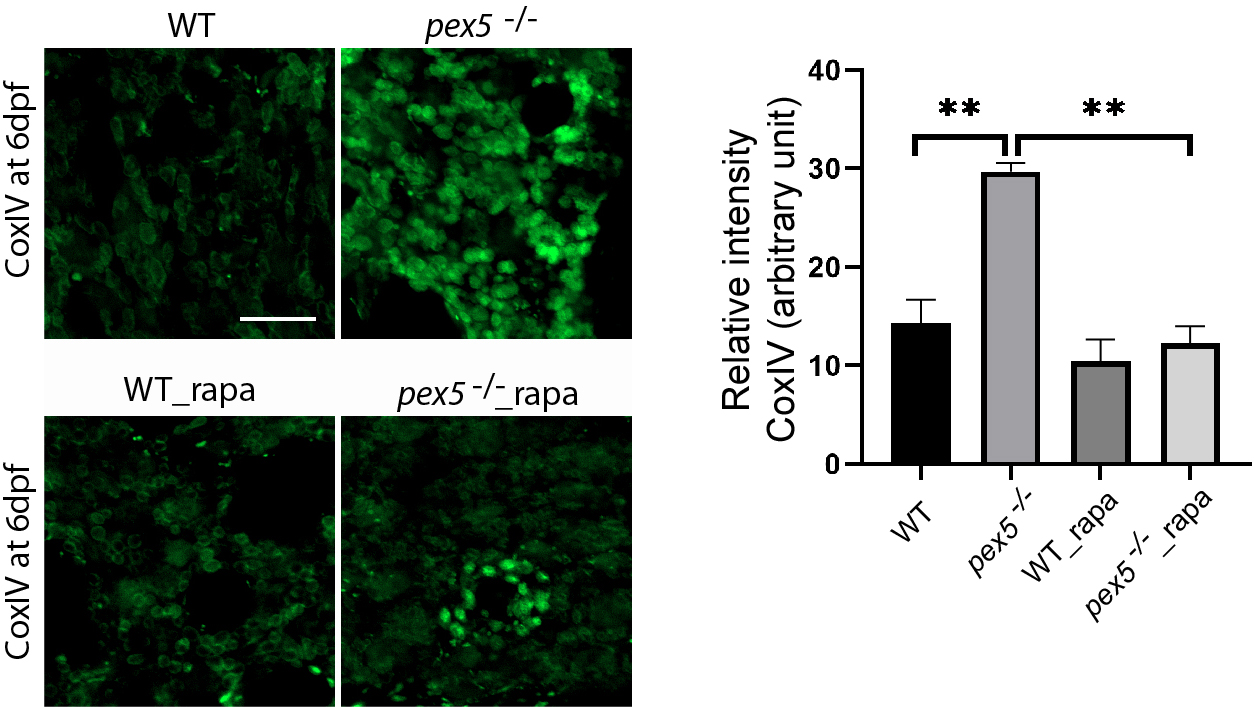


**Supplementary Fig. S6** CoxIV expression in the *pex5^-/-^* liver at 6 dpf is normalized after rapamycin treatment. Endogenous expression of a component of CoxIV is shown following immunofluorescence for comparison between the WT and *pex5*^-/-^ zebrafish liver with or without rapamycin (rapa) treatment. Scale bar = 10 μm. Graphs show quantified signal intensity of CoxIV at the indicated conditions and presented as the average with error bars indicating standard deviation. Statistical significance was determined using the Student t-test in Microsoft Excel; ** indicates p-values < 0.01.


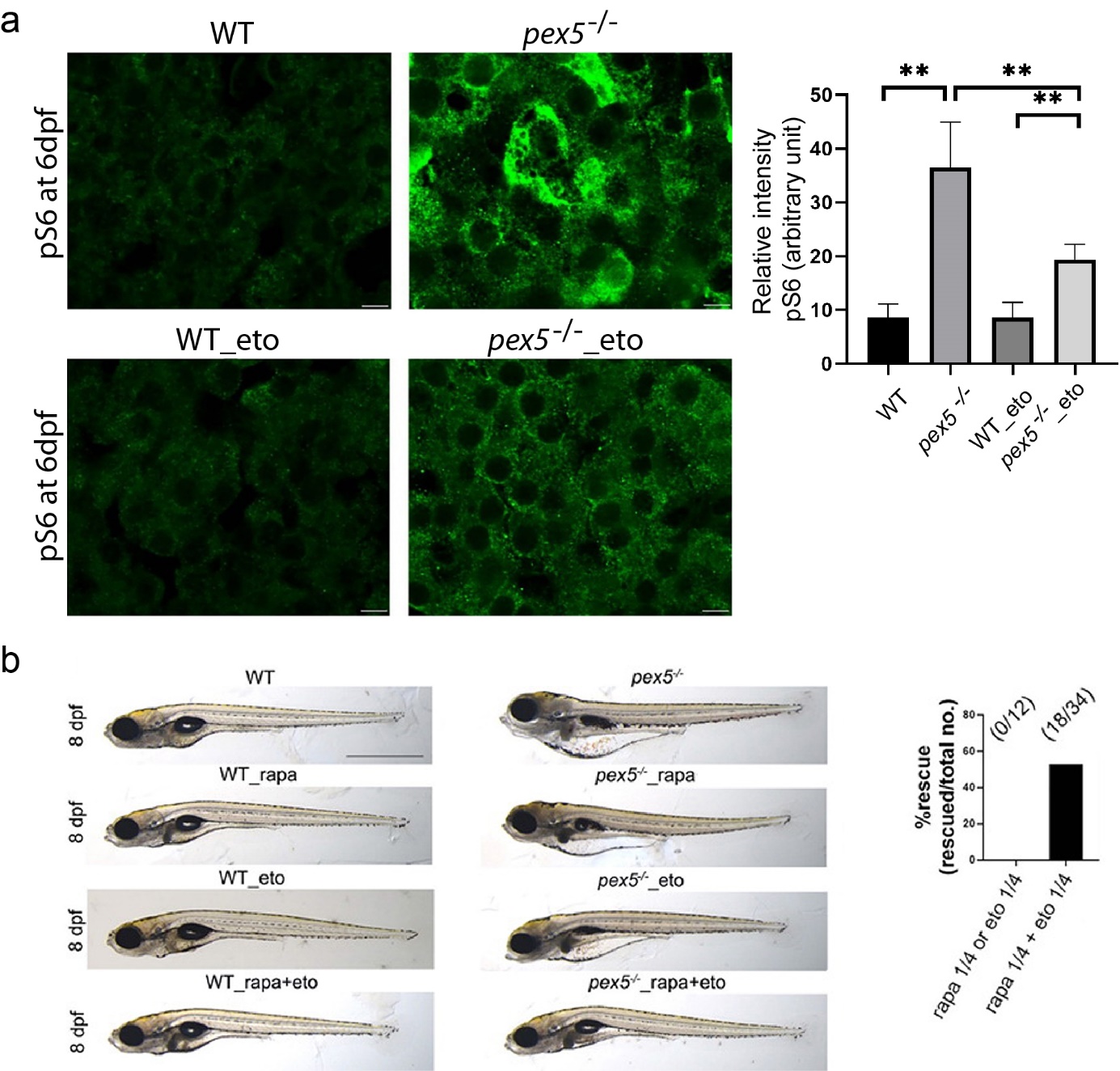


**Supplementary Fig. S7** A positive relationship between mitochondrial function and mTORC1 activity. (a) Etomoxir treatment decreases the level of phosphor-S6, a target of mechanistic target of rapamycin complex 1 (mTORC1), in *pex5*^-/-^ zebrafish. Immunofluorescence was used to detect the phosphorylated form of S6 in the liver sections of WT and *pex5*^-/-^ zebrafish treated with etomoxir (1.5 μM) from 3.5 dpf to 6 dpf. Scale bar = 5 μm. (b) Simultaneous partial repression of mTORC1 and mitochondrial activities rescued the phenotypic severity of the fasted *pex5*^-/-^ zebrafish at 8 dpf. Effect of a combination of rapamycin (rapa 1/4, 62.5 nM) and etomoxir (eto 1/4, 375 nM) or individual drug treated from 3.5 to 8 dpf was tested for their capacity to rescue the fasted *pex5^-/-^* phenotype. A representative larva from each condition was shown laterally, anterior to the left. Scale bar = 1 mm. The graph shows the percentage of near-complete rescue of *pex5*^-/-^ larvae from two independent experiments under the indicated conditions.

**Supplementary Methods**

*Generation of pex5^-/-^ zebrafish.* For designing a guide RNA target vector, two complementary 20 bp oligonucleotides was designed corresponding to *pex5* target sequence and annealed as previously described (1). The correct gRNA vector (Oligo annealed gRNA vector) was selected by digesting the vector with Esp3I. For making nCas9n RNA, pCS2- Cas9 vector was linearized by digesting with Not1 restriction enzyme and purified using a QIAprep column (QIAGEN, Venlo, Netherland). Then nCas9n RNA was synthesized using mMESSAGE mMACHINE SP6 kit (Invitrogen, Waltham, MA, USA) and purified using RNeasy Mini kit (QIAGEN, Venlo, Netherland). For making gRNA, template DNA (*pex5* target oligo annealed gRNA vector) was linearized by digestion with BamHI restriction enzyme and purified using a QIAprep column (QIAGEN, Venlo, Netherland). Then gRNA was synthesized by in vitro transcription using MEGAshortscript T7 kit (Invitrogen, Waltham, MA, USA) and purified using miRNA isolation kit (Invitrogen, Waltham, MA, USA). The mixture of gRNA 20 ng and nCas9n RNA 200 ng was directly injected into one cell stage of embryos derived from wild type cross and grown till adult. The resulting fish were crossed with wild type embryos and genomic sequencing was performed to confirm *pex5* founder (F0) zebrafish. Founder fish was crossed with wild type zebrafish to generate first generation *pex5* heterozygotes which was again confirmed by genomic sequencing.

*Genotyping.* Adult fish or embryos anesthetized using 1X tricaine were subjected to fin clip or tail section, respectively. While being genotyped, fin clipped adult fish were placed in a labeled tank with a fresh water and embryos were placed in a tube with appropriate medium for experimental purpose. Fin clip or tail section was transferred into a tube containing 50 µl DNA digestion buffer (10 mM Tris + 50 mM EDTA + 200 mM NaCl + 0.5% SDS + 0.5 mg/ml Proteinase K + water) and incubated at 55°C overnight. 1 µl of the sample was then diluted with 49 µl nucleotide free water and 1 µl was used for genomic PCR using forward primer 5' GGGAGCCAATCCCCTCATGAAACTG 3' and reverse primer 5' CAGCACATCGTTTGGTCTCAGCCC 3' that were designed to amplify the region that covers deleted base pair of *pex5* gene. Accu Power Taq PCR Pre Mix (Bioneer, Daejean, South Korea) was used for PCR reaction and the reaction was performed according to manufacturer instructions.

*Cryosection of zebrafish embryos*. Embryos fixed with 4% PFA overnight at 4°C were washed with PBST. Embryos were then embedded in the embedding solution (1.5% agarose+ 5% sucrose) at 65°C in inverted position for transverse section or as per required and the agarose was allowed to solidify at room temperature. The agarose embedded embryos were soaked in 30% sucrose solution overnight at 4°C. Then the sample was frozen by placing it on top of methyl butane dipped in liquid nitrogen by using plastic mold and stored in -80°C until needed. The samples were then embedded in OCT compound and sectioned using Thermo Fisher Scientific cryostat (HM525 NX, Waltham, MA, USA) at desired thickness. The sectioned samples were attached to super frost microscopic slides and dried for 1-3 hour and processed as per specific experiments.

*Immunofluorescence.* Section slides after drying for 1-3 hours were washed with 1X PBS three times for 5 minutes. Tissues samples were then incubated in a blocking solution (sheep serum 0.5 ml + 100 mg/ml BSA 0.5ml + 1X PBS to make total of 20ml) for 1 hour at room temperature. Samples were then treated with primary antibody diluted in a blocking solution at 4°C overnight and 1X PBS washing was done for 5 minutes three times followed by secondary antibody (diluted in blocking solution) treatment at room temperature for 2 hours**.** Section slides were then washed with 1X PBS three times for 5 minutes. Tissue samples were then incubated with DAPI (1:1000 in 1X PBS, Roche, Basel, Switzerland) for 10 minutes in the dark followed by PBS washing three times for 5 minutes. Samples were then mounted with cover slip and mounting solution, and images were taken using a confocal microscope (Olympus IX81, Fluoview FV1000). For quantification, sectioned images were chosen and signal intensity of each image was measured using ImageJ (NIH, US). Graphs were generated using Prism 8.0 (GraphPad software, USA) to show average intensity with standard deviation. Statistical significance (p<0.05 or p<0.01) was determined using an unpaired Student-t Test.

*Chemicals, Reagents and Antibodies*. Restriction enzymes, BglII, SalI and NotI and their buffer plus T4 DNA ligase and ligase buffer were purchased from New England Biolab (NEB, Ipswich, MA, USA). Esp31 was purchased from Thermo Fisher Scientific (Waltham, MA, USA). Oil Red-O solution (O1391) and PAS solution (3952016) were from Sigma (St. Louis, MO, USA). In situ cell death detection kit was from Roche (11684817910, Basel, Switzerland). Primary antibodies 4ebp1 (9644S) and p-4ebp1 (2855S) was from Cell Signaling Technology (Danvers, MA, USA); Lc3 (L8918) and N-acetyl-L-cysteine (A8199) were from Sigma (St. Louis, MO, USA); Abcd3 (ab3421), total OXPHOS human WB antibody cocktail (ab110411) and 4-hydroxynonenal (ab46545) were from Abcam (Cambridge, UK); ATP synthase (Complex V) subunit alpha (459240) and Anti-Oxphos Complex IV subunit I (459600) were from Life Technologies (Carlsbad, CA, USA); p62 was from MBL (Japan); Ubiquitin (ADI-SPA-200-D) was from Enzo Life Sciences, Inc. (Farmingdale, NY, USA); and β-actin (Sc-47778) was from Santa Cruz Biotechnology, Inc (Dallas, TX, USA). Secondary antibodies, goat anti-rabbit conjugated to Alexa Fluor 488 (A11034), goat anti-mouse Alexa Fluor 488 (A11001), goat anti-mouse Alexa Fluor 568 (A11031) and goat anti-rabbit Alexa Fluor 568 (A11011), were from Life Technologies (Carlsbad, CA, USA). UK-5099 (Pz0160) and chloroquine diphosphate salt (C6628) were from Sigma (St. Louis, MO, USA). Rapamycin (1292) was from TOCRIS (Bristol, UK).

**Reference**

1. Jao LE, et al. Efficient multiplex biallelic zebrafish genome editing using a CRISPR nuclease system. *Proceedings of the National Academy of Sciences of the United States of America.* 2013;110(34):13904-9.
